# Melanoma cells induce dedifferentiation and metabolic changes in adipocytes present in the tumor niche

**DOI:** 10.1186/s11658-023-00476-3

**Published:** 2023-07-22

**Authors:** Aleksandra Simiczyjew, Justyna Wądzyńska, Katarzyna Pietraszek-Gremplewicz, Magdalena Kot, Marcin Ziętek, Rafał Matkowski, Dorota Nowak

**Affiliations:** 1 Department of Cell Pathology, Faculty of Biotechnology, University of Wroclaw, 50-383 Wroclaw, Poland; 2 Department of Oncology and Division of Surgical Oncology, Wroclaw Medical University, Plac Hirszfelda 12, 53-413 Wroclaw, Poland; 3 Lower Silesian Oncology, Pulmonology, and Hematology Center, Plac Hirszfelda 12, 53-413 Wroclaw, Poland

**Keywords:** Adipocytes, Melanoma, Cancer-associated adipocytes

## Abstract

**Background:**

One of the factors that affect the progression of melanoma is the tumor microenvironment, which consists of cellular elements, extracellular matrix, acidification, and a hypoxic state. Adipocytes are one of the types of cell present in the niche and are localized in the deepest layer of the skin. However, the relationship between fat cells and melanoma remains unclear.

**Methods:**

We assessed the influence of melanoma cells on adipocytes using an indirect coculture system. We estimated the level of cancer-associated adipocyte (CAA) markers through quantitative PCR analysis. The fibroblastic phenotype of CAAs was confirmed by cell staining and western blotting analysis. The lipid content was estimated by lipid detection in CAAs using LipidSpot and by quantitative analysis using Oil Red O. The expression of proteins involved in lipid synthesis, delipidation, and metabolic processes were assessed through quantitative PCR or western blotting analysis. Lactate secretion was established using a Lactate-Glo™ assay. Proteins secreted by CAAs were identified in cytokine and angiogenesis arrays. The proliferation of melanoma cells cocultured with CAAs was assessed using an XTT proliferation assay. Statistical analysis was performed using a one-way ANOVA followed by Tukey’s test in GraphPad Prism 7 software.

**Results:**

Obtained CAAs were identified by decreased levels of leptin, adiponectin, resistin, and FABP4. Adipocytes cocultured with melanoma presented fibroblastic features, such as a similar proteolytic pattern to that of 3T3L1 fibroblasts and increased levels of vimentin and TGFβRIII. Melanoma cells led to a reduction of lipid content in CAAs, possibly by downregulation of lipid synthesis pathways (lower FADS, SC4MOL, FASN) or enhancement of lipolysis (higher level of phosphorylation of ERK and STAT3). Adipocytes cocultured with melanoma cells secreted higher IL6 and SerpinE1 levels and produced less CCL2, CXCL1, and angiogenic molecules. CAAs also showed metabolic changes comprising the increased secretion of lactate and enhanced production of glucose, lactate, and ion transporters. In addition, changes in adipocytes observed following melanoma coculture resulted in a higher proliferation rate of cancer cells.

**Conclusions:**

Melanoma cells led to decreased lipid content in adipocytes, which might be related to enhanced delipidation or reduction of lipid synthesis. Fibroblast-like CAAs showed metabolic changes that may be the reason for accelerated proliferation of melanoma cells.

**Supplementary Information:**

The online version contains supplementary material available at 10.1186/s11658-023-00476-3.

## Background

Melanoma is a very invasive type of cancer that rapidly spreads throughout an organism. Its treatment consists of surgical removal of the lesion and systemic treatment if metastasis has occurred [1]. Unfortunately, therapy is often not effective because resistance to the administered drugs appears rapidly. One of the reasons for this phenomenon might be interactions between melanoma cells and elements of the surrounding tumor microenvironment (TME). The tumor niche contains many types of cells, including cancer-associated fibroblasts (CAFs), keratinocytes, cancer-associated adipocytes (CAAs), and immune cells. The TME also consists of the extracellular matrix (ECM) that fills the space between the cells and the molecules secreted by neighboring cells, which may affect the effectiveness of the cancer treatment [2].

Adipose tissue constitutes the predominant component of the deepest layer of the skin, the hypodermis, and is composed mainly of adipocytes [3]. The major function of differentiated adipocytes is the accumulation of lipids (mostly triglycerides), which can later be released in the form of free fatty acid (FFA) into the environment. Apart from its role in lipid storage, adipose tissue is also involved in inflammatory processes and hormone secretion [4, 5]. Adipocytes are known to release components called adipokines, which include inflammatory factors [interleukin 6 (IL-6), interleukin 8 (IL-8), C–C motif chemokine ligand 2 (CCL2), C-X-C motif chemokine ligand 1 (CXCL1), pentraxin-related protein (PTX3), and plasminogen activator inhibitor-1(PAI-1)]. They also release metabolic markers [insulin-like growth factor-binding protein (IGFBP)], angiogenic factors [angiopoietin-1, angiogenin, vascular endothelial growth factor (VEGF), and insulin-like growth factor 1 (IGF-I)], and hormones (leptin, resistin, adiponectin) [2, 6]. There are receptors on the surfaces of cancer cells that can bind the majority of these molecules [7].

Adipokines are involved in the activation of several signaling pathways, including PI3K (phosphoinositide 3-kinase)/AKT (protein kinase B), MAPK (mitogen-activated protein kinase), and JAK (Janus kinase)/STAT (signal transducer and activator of transcription). These pathways activate the expression of genes that encode proteins involved in tumor progression and thus support the proliferation, invasion, and resistance to apoptosis of cancer cells [6, 8]. The presence of adipocytes, especially in obese patients with cancer, is associated with less effective treatment and a higher rate of cancer-related deaths [6]. Current reports indicate a positive correlation between body fat accumulation and the risk of melanoma progression [9, 10]. Obesity also promotes the growth of melanoma tumors and the appearance of metastases in the lungs and lymph nodes in mice [11–13].

Furthermore, a high level of leptin in serum is associated with increased risk of melanoma in humans, while in mice, it is related to greater weight and size of melanoma tumors [11, 14]. Leptin and resistin are both secreted by adipocytes and affect synthesis of FASN (fatty acid synthase) and the activation of the AKT-based signal transduction pathway, thus promoting the proliferation of melanoma cells [8, 15]. Molecules released by adipocytes also include a source of energy and structural components that are essential for the rapid proliferation of cancer cells.

Metabolic changes that cancer cells trigger in adipocytes result in increased lipolytic activity and the release of fatty acids into microenvironment. These processes lead to the appearance of cachexia in patients with cancer. This phenomenon often appears in patients with cancer in advanced stages and it is connected to the loss of adipose tissue. This loss is associated with increased lipolysis, disturbances in the appropriate storage of FFA and triacylglycerol, as well as glycerol release [16, 17].

It was recently demonstrated that adipocytes can directly transfer lipids to melanoma cells in vivo [18]. Moreover, elevated lipid content in melanoma in comparison to melanocytes is associated with lipid uptake rather than the increase in de novo lipogenesis. The level of fatty acids is increased in melanoma cells that have been cocultured with adipocytes and is linked to increased proliferation of cancer cells, activation of the PI3K/AKT signaling pathway, and changes in the distribution of cell cycle phases [19]. The migration of these cells is also increased, which is connected to the activation of the Wnt pathway. The invasion of melanoma cells triggered by the adipocyte-conditioned medium is related to the expression of the epithelial–mesenchymal transition-associated genes in cancer cells [20, 21].

Moreover, adipocytes can stimulate the process of tumor angiogenesis. Coelho et al. indicated that adipocyte-conditioned media containing proangiogenic factors like hepatocyte growth factor and VEGF induced vascular mimicry in melanoma [6]. The influence of adipocytes on melanoma progression is not fully understood yet, but it has been described to a much greater extent than the impact of melanoma on the biology of adipocytes that are present in its surroundings. Obesity is a very serious and constantly growing problem and can promote the invasiveness of melanoma cells. Thus, gaining knowledge about the changes in adipocytes under the influence of melanoma may lead to new therapeutic targets among TME elements or improve current diagnostic methods.

In our previous research, we established an effect of melanoma cells on the biology of fibroblasts and keratinocytes in the tumor niche [22, 23]. Cancer-associated fibroblasts exhibited elevated motility, proteolytic activity, and secretion of lactate, several cytokines, and proteins related to angiogenesis [22]. HaCaT keratinocytes under the influence of melanoma cells exhibited properties of less differentiated cells and tended to form connections with cancer cells rather than adjacent keratinocytes. They also demonstrated increased proteolytic activity, reduced expression of TIMPs (tissue inhibitor of metalloproteinases), and upregulation of ERK activity [23]. Thus, the present study focuses on the influence of melanoma cells on adipocytes’ features, which is not yet fully understood.

## Methods

### Cell culture

Adipocytes were obtained from 3T3L1 mouse preadipocytes [American Type Culture Collection (ATCC), Manassas, Virginia, USA] by differentiation according to the protocol described by Zebisch [24]. Briefly, 2 days after reaching confluence, cells were treated with a mixture of rosiglitazone (Merck, Darmstadt, Germany), insulin (Sigma Aldrich, Burlington, Massachusetts, USA), dexamethasone (Sigma Aldrich, Burlington, Massachusetts, USA), and 3-isobutyl-1-methylxanthine (IBMX; Merck, Darmstadt, Germany) for 48 h. They were then incubated with insulin for another 48 h.

The influence of melanoma cells on adipocytes was assessed using four melanoma cell lines: A375 (CRL-1619) and Hs294T (HTB-140), obtained from the ATCC, and WM1341D (WM1341D-01-0001) and WM9 (WM9-01-0001) cell lines purchased from Rockland Immunochemicals, Inc. (Royersford, Pennsylvania, United States). Adipocytes and melanoma cells were cultured in DMEM medium containing 4.5 g/l of glucose and 1.5 g/l of NaHCO_3_ (IITD PAN, Wrocław, Poland) supplemented with 2 mM glutamine (Thermo Fisher, Waltham, Massachusetts, United States) and antibiotic–antimycotic solution (10,000 U/ml penicillin, 10 mg/ml streptomycin, 25 µg/ml amphotericin B; Thermo Fisher, Waltham, Massachusetts, USA). Moreover, 10% bovine calf serum (BCS) (Sigma Aldrich, Burlington, Massachusetts, USA) was added to the adipocyte culture medium, whereas the culture medium for melanoma cells contained 10% fetal bovine serum (FBS) (Thermo Fisher, Waltham, Massachusetts, USA). Cells were cultured in 25-cm^2^ tissue culture flasks (VWR, Radnor, Pennsylvania, USA) at 37 °C in air containing 5% CO_2_ at 95% humidity. They were passaged twice a week using 0.25% trypsin/0.05% EDTA solution (IITD PAN, Wrocław, Poland).

### Cancer-associated adipocyte acquisition

**Fig. 1:**
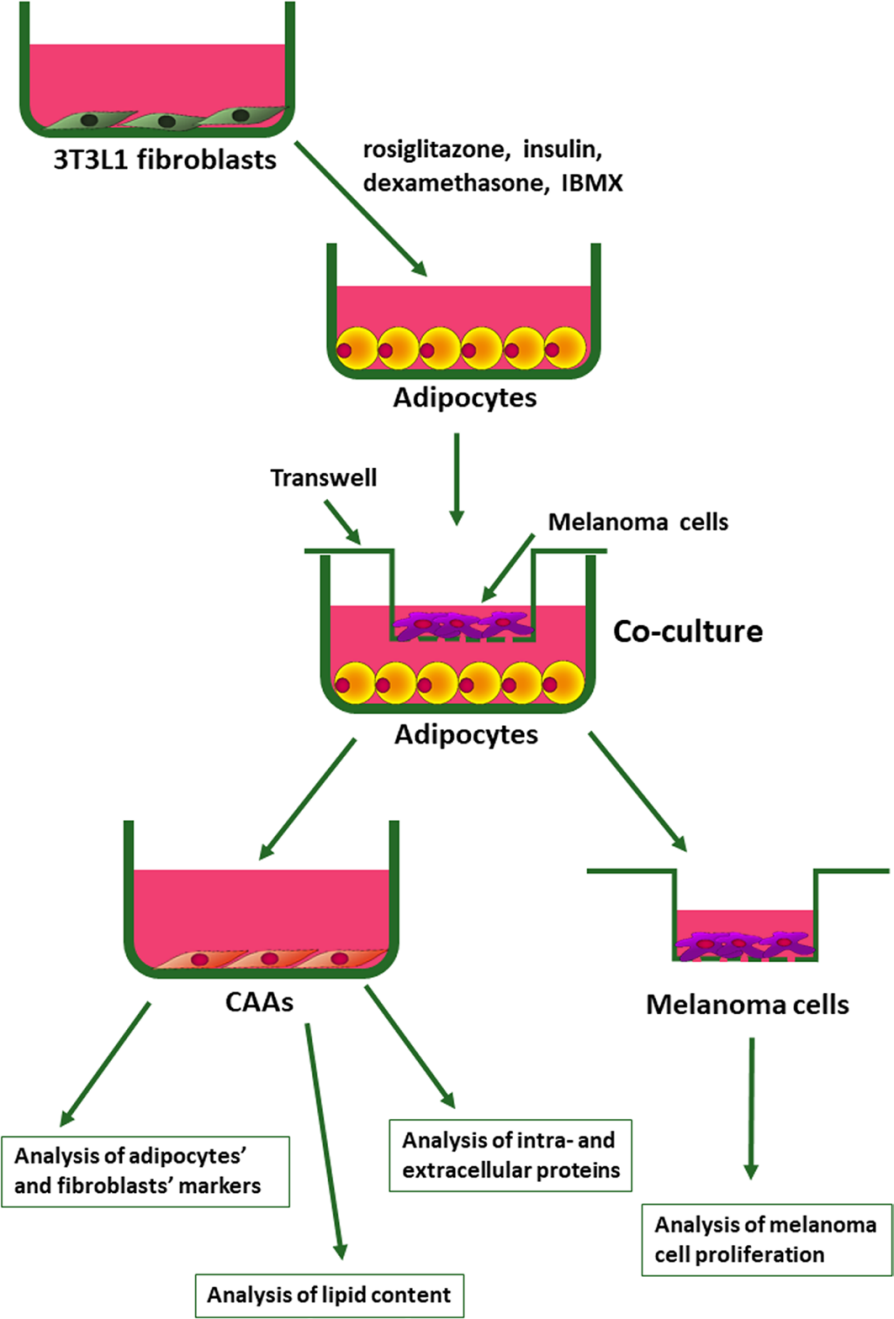
CAA acquisition method. Adipocytes were obtained from 3T3L1 mouse preadipocytes by differentiation based on cell treatment with a mixture of rosiglitazone, insulin, dexamethasone, and 3-isobutyl-1-methylxanthine (IBMX) for 48 h, followed by incubation with insulin for another 48 h. Then, adipocytes were cultured for 7 days in the presence of melanoma cells seeded on Transwell inserts in a combination of DMEM with 10% BCS and DMEM with 10% FBS (1:1 ratio). The 3T3L1 fibroblasts and control adipocytes were also grown in such a medium. Next, cells were harvested and used for further experiments, or the medium was changed for DMEM without serum, collected after 72 h, and then used for analysis of extracellular proteins level. Additionally, the proliferation of melanoma cells after coculture with adipocytes was examined

To obtain CAAs, adipocytes were obtained from 3T3L1 preadipocytes and grown for 7 days with melanoma cells on Transwell inserts (0.4 µm pores, Falcon, Corning, New York, USA). During this period, the medium was changed two times (Fig. 1). After incubation, CAAs were collected and used for further experiments. In indirect coculture, the used media had to be suitable for two different types of cells. Therefore, adipocytes and melanoma cells were grown in a combination of DMEM with 10% BCS and DMEM with 10% FBS (1:1 ratio). Therefore, the control for each experiment constituted adipocytes cultured in such a medium. We compared the results obtained for CAAs to those obtained for control adipocytes and non-differentiated 3T3L1 fibroblasts.

### Real-time (RT)–PCR

To measure the expression level of selected genes in CAAs, RNA was isolated using an RNA purification kit (EURx, Gdańsk, Poland) according to the manufacturer’s instructions. Reverse transcription reaction was conducted using a High-Capacity cDNA Reverse Transcription Kit (Applied Biosystems, Waltham, Massachusetts, USA) by following the manufacturer’s protocol. Quantitative PCR was performed using PowerUp™ SYBR™ Green Master Mix on a StepOnePlus system (Applied Biosystems, Waltham, Massachusetts, USA). The specificity of the primers was verified at different temperatures using melting curves. The results were normalized to EEF2 expression based on the comparative threshold cycle value (CT ) method (ΔCT = 2^−(CT gene of interest − CT housekeeping gene)^. Three biological repetitions were each performed in duplicate. Sequences of the utilized primers are shown in Table 1.

**Table 1:** Sequences of utilized primers

Gene		Forward primer 5’-3’	Reverse primer 5’-3’
Abbreviation	Full name of the gene		
*Adiponectin*	–	aatcttgcccagtcatgccg	ccttaggaccaagaagacctgc
*Leptin*	–	gagacccctgtgtcggttc	ggaatgaagtccaagccagtgac
*EEF2*	Eukaryotic translation elongation factor 2	gacatcaccaagggtgtgcag	tcagcacactggcataggc
*FABP4*	Fatty acid binding protein	gcttgtctccagtgaaaacttcg	ccagtttgaaggaaatctcggtg
*Resistin*	–	caagacttcaactccctgtttcc	ggaaaccacgctcacttccc
*SC4MOL*	Sterol-C4-methyl oxidase-like protein	gtgttggcgtgttcagctctg	agatggcttcgtgaactatcaggg
*PLIN1*	Perilipin 1	ctccagagagttctgcagctg	caactcattggcagctgtgaac
*FADS1*	Fatty acid desaturase 1	cgccaaacgcgctactttacttg	catcctgacccgcgtagtgg
*MCT1*	Monocarboxylate transporter 1	cctatgcatttcccaaatccatc	gatactgctgataggacctcc
*NHE1*	Sodium–hydrogen antiporter 1	ctcatcgcctcaggagtag	ggtgctgatgacgaaggtc
*GLUT1*	Glucose transporter 1	gatcactgcagttcggctataac	cctgccaaagcgattaacaaag

### Western blot analysis

Cocultured adipocytes were transferred on ice and washed three times with PBS. Lysates were harvested by the addition of urea buffer (50 mM Tris, pH 7.4, 5% SDS, 8.6% sucrose, 74 mM urea, 1 mM dichlorodiphenyltrichloroethane) supplemented with protease and phosphatase inhibitors cocktails (Sigma Aldrich, Burlington, Massachusetts, USA). The protein concentration in lysates was determined using a standard bicinchoninic acid (BCA) procedure (Thermo Fisher, Waltham, Massachusetts, USA). Samples of identical amounts of protein (10 μg) were separated by 10% polyacrylamide gel electrophoresis in the presence of sodium dodecyl sulfate (SDS-PAGE) as reported by Laemmli [25] and then transferred to nitrocellulose membranes as reported by Towbin et al. [26].

Primary antibodies directed against FASN (Santa Cruz Biotechnology, Dallas, Texas, USA sc-48357, 1:2000), FABP4 (Cell Signaling, Danvers, Massachusetts, United States #2120, 1:1000), vimentin (GeneTex, Irvine, California, USA GTX100619, 1:500), TGFRβIII (Cell Signaling, Danvers, Massachusetts, USA #2519, 1:1000), pERK1/2 (Cell Signaling, Danvers, Massachusetts, USA sc-9101, 1:1000), ERK1/2 (Cell Signaling, Danvers, Massachusetts, USA sc-9102, 1:1000), STAT3 (Santa Cruz Biotechnology, Dallas, Texas, USA sc-8019, 1:200), and pSTAT3 (Santa Cruz Biotechnology, Dallas, Texas, USA sc-8059, 1:200), as well as goat anti-rabbit and anti-mouse secondary antibodies conjugated with horseradish peroxidase (Cell Signaling, Danvers, Massachusetts, USA 1:4000), were utilized according to the manufacturer’s protocols. Immunoblots were developed using Clarity Western ECL Substrate (Bio-Rad, Hercules, California, USA) or Clarity Max Western ECL Substrate (Bio-Rad, Bio-Rad, Hercules, California, USA). They were then scanned with ChemiDoc (Bio-Rad, Bio-Rad, Hercules, California, USA) and analyzed with ImageLab Software (ver. 6.0, Bio-Rad, Bio-Rad, Hercules, California, USA). At least three independent experiments were conducted, and the results were normalized to Ponceau S staining.

### Fluorescent-gelatin degradation assay

Fluorescent-gelatin degradation assays were performed according to the protocol described by Mazurkiewicz et al. [27]. Coverslips coated with poly-L-lysine (Corning, New York, USA) were washed with PBS and then fixed with 0.5% glutaraldehyde for 15 min. Next, the coverslips were placed on a 30-μl drop of gelatin labeled with fluorescein (Invitrogen, Waltham, Massachusetts, USA), incubated for 10 min, and rinsed with PBS. Then, 5 mg/ml sodium borohydride (Merck, Darmstadt, Germany) was used for quenching the residual reactive groups.

After washing with PBS, cells were seeded in 24-well plates containing the prepared coverslips and incubated at 37 °C in air with 5% CO_2_ and 95% humidity. After 16 h, cells were fixed with 4% formaldehyde, permeabilized with 0.1% Triton X-100, and stained with Alexa Fluor 568 phalloidin (Thermo Fisher, Waltham, Massachusetts, USA) to visualize filamentous actin. Confocal images were captured using a Leica Stellaris 8 (Leica, Wetzlar, Germany) and LAS X software (ver. 3.3.0, Leica, Wetzlar, Germany). Locations of degraded gelatin were visible as dark areas that lack fluorescence in the bright-green fluorescent gelatin matrix. Experiments were conducted with three repetitions.

### Cytochemistry

The subcellular distribution of cell nuclei and lipid droplets was examined by fluorescence. After 7 days of coculture, cells were fixed with 4% formaldehyde and permeabilized with 0.1% Triton X-100 in PBS. Unspecific binding sites were then blocked with 1% bovine serum albumin (Sigma Aldrich, Burlington, Massachusetts, USA). LipidSpot 488 (Biotium, Fremont, California, USA) was applied to visualize lipid droplets present inside the cells. Cell nuclei were visualized with Hoechst 33342 reagent (Invitrogen, Waltham, Massachusetts, USA). Then, coverslips were mounted with Dako fluorescent mounting medium (Agilent, Santa Clara, California, USA). For each condition, representative cells are shown. Experiments were conducted with three repetitions.

### Oil Red O staining

An Oil Red O staining assay was performed to determine the amount of neutral lipids within cells. The adipocytes were seeded on six-well plates. After 7 days of coculture with melanoma cells, adipocytes, and 3T3L1 fibroblasts, they were fixed at room temperature for 30 min with 4% formaldehyde, washed with PBS and water, incubated with 60% isopropanol for 5 min, and then stained with Oil Red O (Sigma Aldrich, Burlington, Massachusetts, USA). This was done at room temperature for 20 min using a working solution containing three parts by volume of 0.5% (w/v) stock solution of Oil Red O in isopropanol with two parts of distilled water. After washing three times with water, 100% isopropanol was added, and then the absorbance of Oil Red O was measured at 490 nm using a microplate reader. Experiments were performed with three biological repetitions, and each sample was examined in triplicate.

### Lactate secretion

The level of lactate secreted by CAAs was evaluated using a Lactate-Glo™ Assay (Promega, Madison, Wisconsin, USA). In this test, lactate present in the medium undergoes oxidation catalyzed by lactate dehydrogenase, which is coupled with the reduction of NAD^+^. NADH was then used in proluciferin for luciferin reduction to produce chemiluminescence. The analyzed media were collected after 24 h of CAA incubation in DMEM medium without serum. Experiments were performed according to the manufacturer’s protocol. Luminescence was measured using a GloMax Discover plate reader (Promega, Madison, Wisconsin, USA), and the luminescence value acquired from the control cells’ medium was set as 100%. Experiments were performed with three biological repetitions, and each sample was examined in triplicate.

### Cytokine and angiogenesis arrays

We investigated the composition of the CAA secretome using Proteome Profiler Human Cytokine and Angiogenesis Array Kits (R and D Systems, Minneapolis, Minnesota, USA). The tests allow detection of 36 or 55 different cytokines and chemokines or angiogenesis-related proteins, respectively, thanks to spots of antibodies on nitrocellulose membrane. The experiment was conducted according to the manufacturer’s protocol. Briefly, conditioned media collected from control adipocytes, and adipocytes cocultured with WM1341D, A375, and WM9 cells were mixed with biotinylated detection antibodies. These mixes were then dropped on nitrocellulose membranes containing primary antibody dots and incubated overnight. Next, the membranes were washed, and the signal was detected using streptavidin-HRP. The chemiluminescence signal was measured using a ChemiDoc Imaging System (Bio-Rad, Hercules, California, USA) and analyzed with ImageLab software (Bio-Rad, Hercules, California, USA). Densitometric values were background corrected and then normalized to the mean of reference spots for each membrane.

### Proliferation assay

A Cell Proliferation Kit II (XTT) (Roche, Basel, Switzerland) was used according to the manufacturer’s protocol. Melanoma cells that had been cocultured with CAAs were seeded in 96-well plates. XTT-labeling mixture was added to parallel samples at time 0 (T0) and after 48 h (T48) of cell growth. After 3 h of incubation at 37 °C, the absorbance at 450 nm was measured, and the obtained values were background corrected. Based on the absorbance, the proliferation ratio was calculated by dividing T48 by T0. The control cells’ proliferation was set as 100%. The experiments were conducted with at least three biological repetitions in triplicate for each condition.

### Statistical analysis

All data are given as the means ± standard deviation (SD), and their significance was evaluated with GraphPad Prism 7 software using a one-way ANOVA followed by Tukey’s test.

## Results

### CAA acquisition and identification

To obtain cancer-associated adipocytes, we differentiated 3T3L1 preadipocytes into adipocytes using the protocol described by Zebisch et al. [24] and then cocultured them with melanoma cells present on the Transwell inserts (Fig. 1). To verify the effects of primary and metastatic melanoma cells on adipocytes, we performed experiments using four different melanoma cell lines of differing origin. Two of them were derived from primary tumors (WM1341D and A375), and the others (WM9 and Hs294T) were isolated from melanoma metastases.

**Fig. 2:**
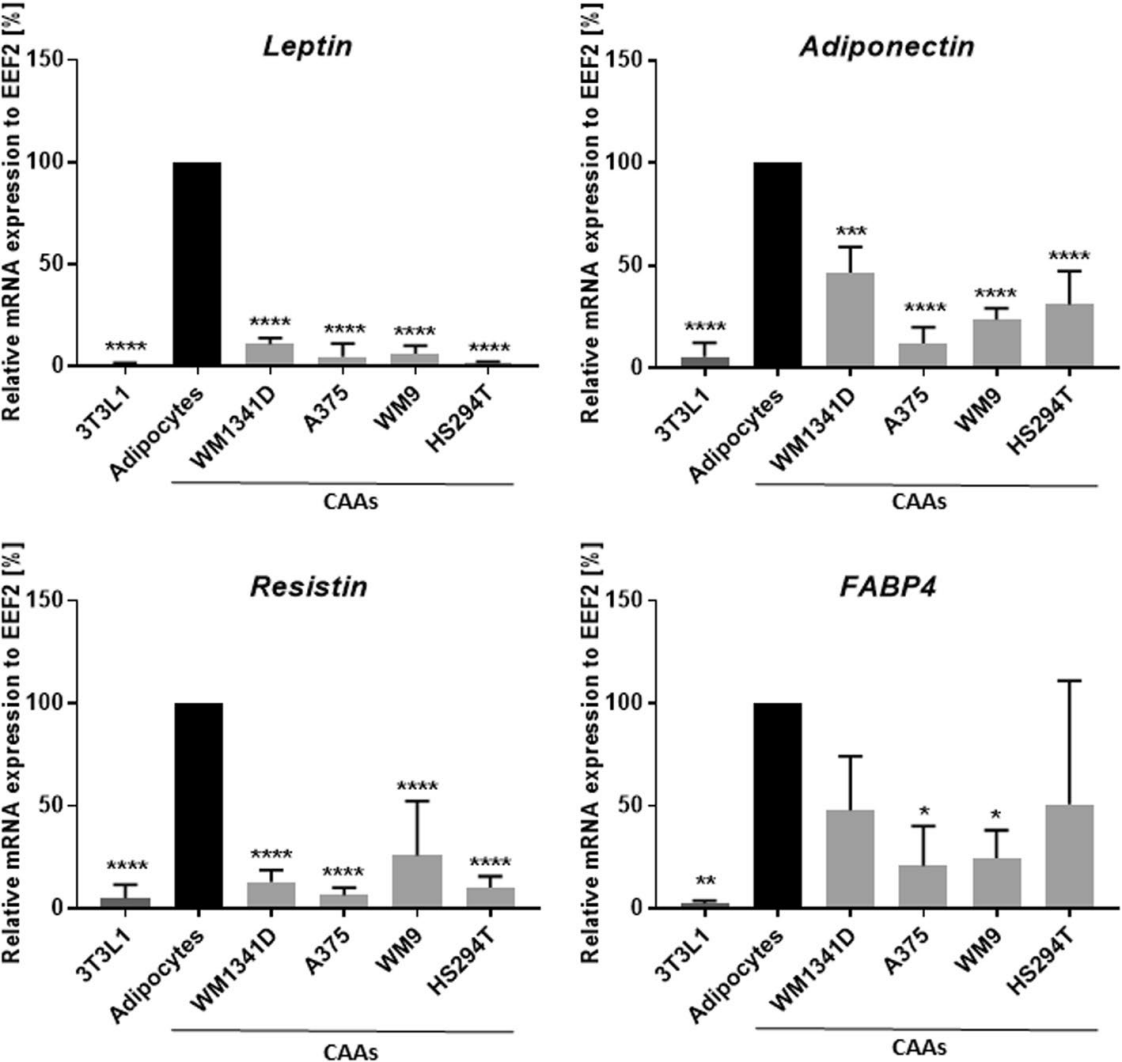
Characterization of cancer-associated adipocytes (CAAs). Adipocytes were cocultured with melanoma cells (lines: WM1341D, A375, WM9, and Hs294T) present on Transwell inserts, and then the expression of leptin, adiponectin, resistin, and FABP4 was established in obtained CAAs, as well as nondifferentiated 3T3L1 fibroblasts and control adipocytes, using real-time PCR analysis. For quantification, the level of expression was normalized to the expression of EEF2 and presented as the percent of control (untreated adipocytes). The mean of at least three biological repetitions ± standard deviation (SD) is shown. Asterisks indicate statistically significant differences between control adipocytes and 3T3L1/CAAs. The significance levels were set at *p* ≤ 0.05 (*), *p* ≤ 0.01 (**), *p* ≤ 0.001 (***), or *p* ≤ 0.0001 (****). *FABP4* fatty acid-binding protein 4, *EEF2* eukaryotic translation elongation factor 2

Next, we assessed the transformation of adipocytes into cancer-associated adipocytes based on typical markers for differentiated adipocytes. Moreover, we verified whether the obtained CAAs present features of dedifferentiation into fibroblasts, which can occur under the influence of cancer cells [28]. Therefore, the results obtained for CAAs were compared not only with control adipocytes, but also with 3T3L1 fibroblasts. We examined whether adipocytes cocultured with melanoma cells demonstrate decreased expression of adipocyte differentiation markers such as leptin, resistin, adiponectin, and fatty acid binding protein (FABP4) by assessing the expression of these genes in the cells (Fig. 2).

The choice of the tested molecules was related to the fact that they occur in adipocytes, while in CAAs their level may be reduced. Leptin, adiponectin, and resistin are adipokines produced by mature adipocytes. FABP4 is used as a marker for differentiated adipocytes [29]. We noticed an increased level of all tested molecules in control adipocytes in comparison with the original 3T3L1 fibroblasts, which means that the obtained adipocytes were fully differentiated and mature. Moreover, we demonstrated that the expression of all of these genes was reduced in cancer-associated adipocytes in comparison to the control. This effect was evident in CAAs, regardless of whether it was induced by primary or metastatic melanoma cell lines (Fig. 2).

### Fibroblastic features of CAAs

Proteolytic enzymes released by cells present in the tumor niche are engaged in the digestion of ECM elements and the formation of a path that facilitates invasion of cancer cells through the tissues. To evaluate the CAAs’ proteolytic activity, a gelatin–FITC degradation assay was performed. In this method, fluorescently-labeled gelatin is digested by proteases secreted by the cells, resulting in the appearance of black spots on a green fluorescent background. Furthermore, phalloidin conjugated to Alexa Fluor 568 was used to stain filamentous actin (F-actin) to visualize the cell shape and actin cytoskeleton organization.

**Fig. 3:**
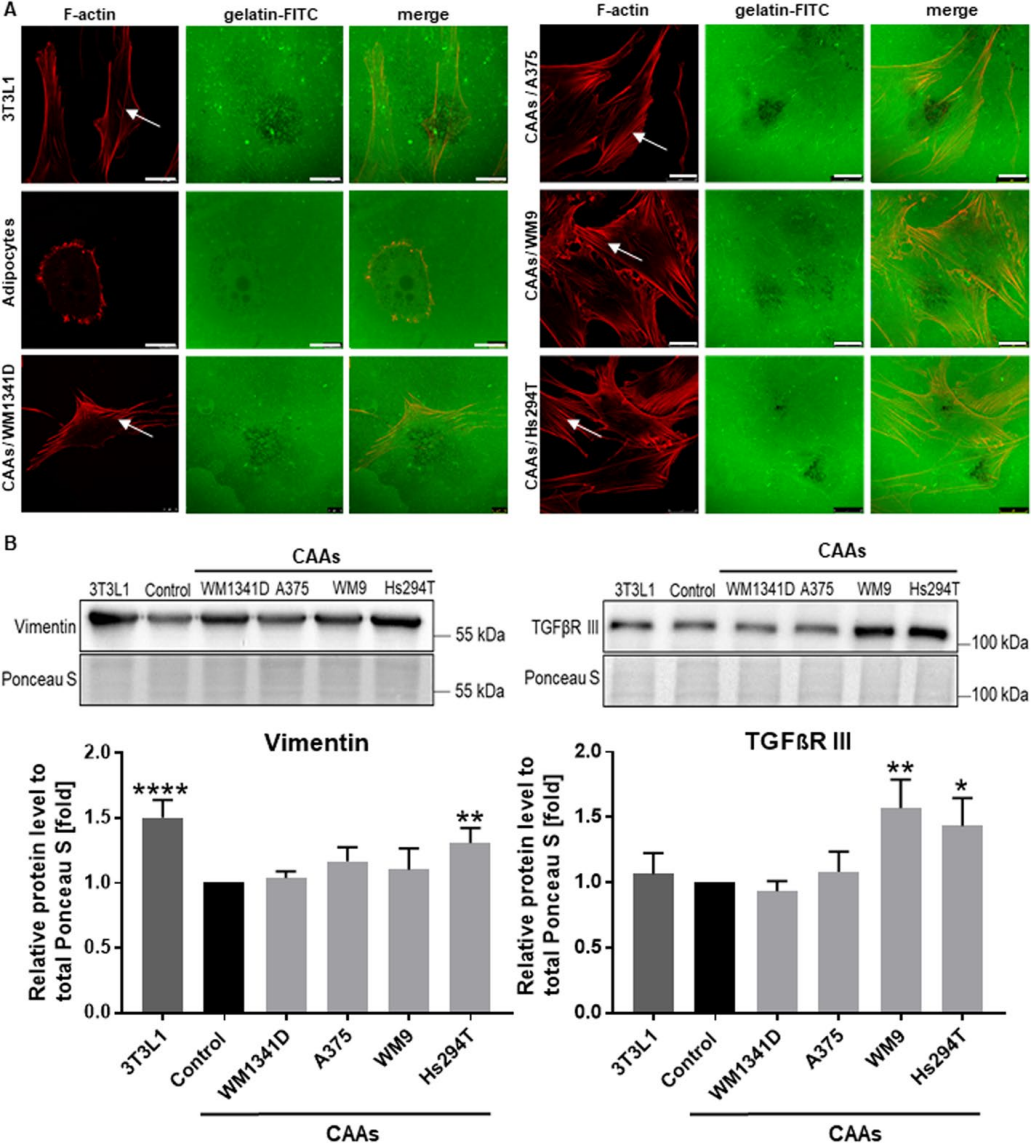
Features of fibroblasts exhibited by CAAs. **A** Adipocytes were cocultured with melanoma cells ( lines: WM1341D, A375, WM9 and Hs294T) present on Transwell inserts and then obtained CAAs as well as the non-differentiated 3T3L1 fibroblasts and control adipocytes were seeded on coverslips coated with gelatin-FITC (green). After 16 h of incubation, cells were fixed and stained using phalloidin-Alexa Fluor 568 to visualize F-actin (red). Areas of gelatin degradation are visible as dark holes on a green background. White arrows indicate stress fibers. Scale bar: 25 μm. **B** Western blot analysis of protein level of vimentin and TGFβ receptor III (TGFβRIII) in cell lysates of 3T3L1 cells; control adipocytes, and adipocytes cultured with melanoma cells growing on Transwell inserts. The signal was normalized to total protein content assessed by Ponceau S staining. The mean of at least three biological repetitions ± SD is shown. Asterisks indicate statistically significant differences between control adipocytes, 3T3L1 fibroblasts and CAAs. The significance levels were set at *p* ≤ 0.05 (*), *p* ≤ 0.01 (**), or *p* ≤ 0.0001 (****).

We detected different proteolysis patterns in 3T3L1 fibroblasts and differentiated adipocytes (Fig. 3A). In the case of 3T3L1 fibroblasts, the patterns appeared more point-like and were diffused, while in adipocytes, locations of digestion were rather circular. Moreover, the proteolysis pattern of CAAs was more similar to that presented by normal fibroblasts than to that of control adipocytes (Fig. 3A). This suggests that adipocytes under the influence of melanoma cells acquire features that are typical of fibroblasts. Analysis of the CAAs’ actin cytoskeleton organization also indicated their significant similarity to fibroblasts. These cells were characterized by a spindle-shaped morphology and a large number of stress fibers (Fig. 3A, white arrows), analogously to 3T3L1 cells. However, adipocytes exhibited a rounded shape and poorly organized actin skeleton that was devoid of stress fibers.

We also assessed the level of proteins that are typically present in fibroblasts. Vimentin is a marker of fibroblasts, while TGFβ receptor type III (TGFβRIII) functions as a coreceptor that participates in the binding of TGFβ on the surface of fibroblasts [30]. Both vimentin and TGFβRIII levels were increased in 3T3L1 fibroblasts and CAAs in comparison with control adipocytes (Fig. 3B, Additional file 1). This difference was statistically significant for these two proteins of Hs294T cocultured CAAs, whereas the change in TGFβRIII level was statistically significant in the case of CAAs cocultured with both metastatic cell lines (WM9 and Hs294T).

### Delipidation of CAAs

**Fig. 4:**
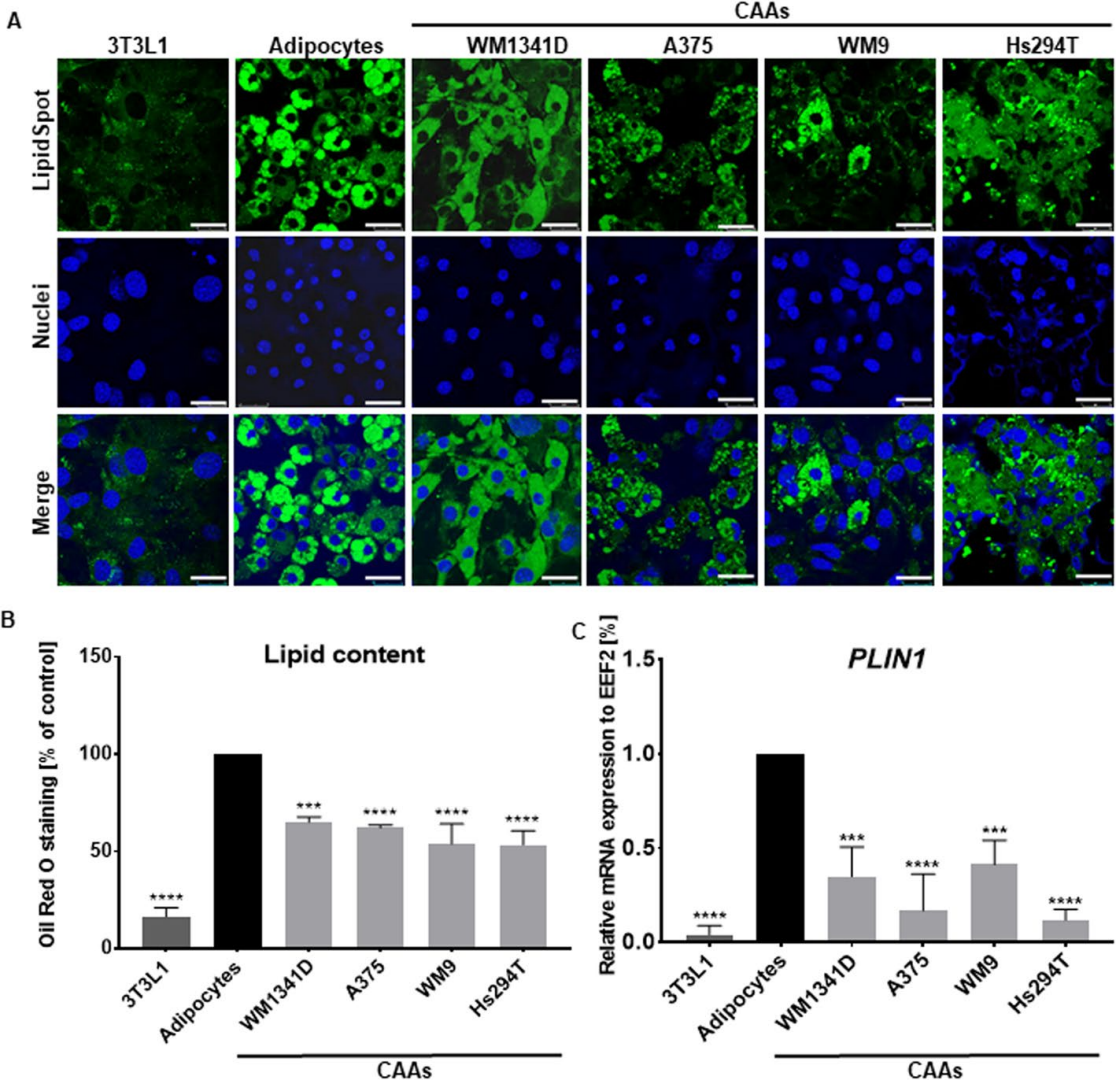
Loss of lipid content in cancer-associated adipocytes. Adipocytes were cocultured with melanoma cells (lines: WM1341D, A375, WM9, and Hs294T) present on Transwell inserts. **A** After coculture, obtained CAAs, control adipocytes, and 3T3L1 fibroblasts were seeded on coverslips, fixed, and stained with Lipid Spot 488 to visualize lipid droplets. Cell nuclei were stained using Hoechst 33,342 reagent. Scale bar: 25 μm. **B** To quantify the amount of neutral lipids, cells were fixed and then stained with Oil Red O, and the amount was measured spectrophotometrically. **C** Expression of PLIN1 (perilipin1) in 3T3L1 cells, control adipocytes, and CAAs established by real-time PCR analysis. For quantification, the samples were normalized against the expression of EEF2. For all experiments, the control constitutes normal adipocytes. In the case of quantitative analyses, the mean of at least three biological repetitions ± SD is shown. Asterisks indicate statistically significant differences between control cells and 3T3L1/CAAs. The significance levels were set at *p* ≤ 0.01 (**), *p* ≤ 0.001 (***), or *p* ≤ 0.0001 (****)

It was demonstrated earlier that delipidation of adipocytes can occur under the influence of melanoma [3]. We also observed a decrease in the number and size of lipid droplets in all tested CAAs compared with control adipocytes (Fig. 4A). Qualitative data obtained from staining with Lipid Spot 488 were confirmed quantitatively by spectrophotometric measurement of the amount of lipids in cells stained with Oil Red O (Fig. 4B). Moreover, CAAs had lower expression of the gene encoding perilipin 1, a protein involved in lipid droplet formation and maintenance, in comparison to control adipocytes (Fig. 4C).

**Fig. 5:**
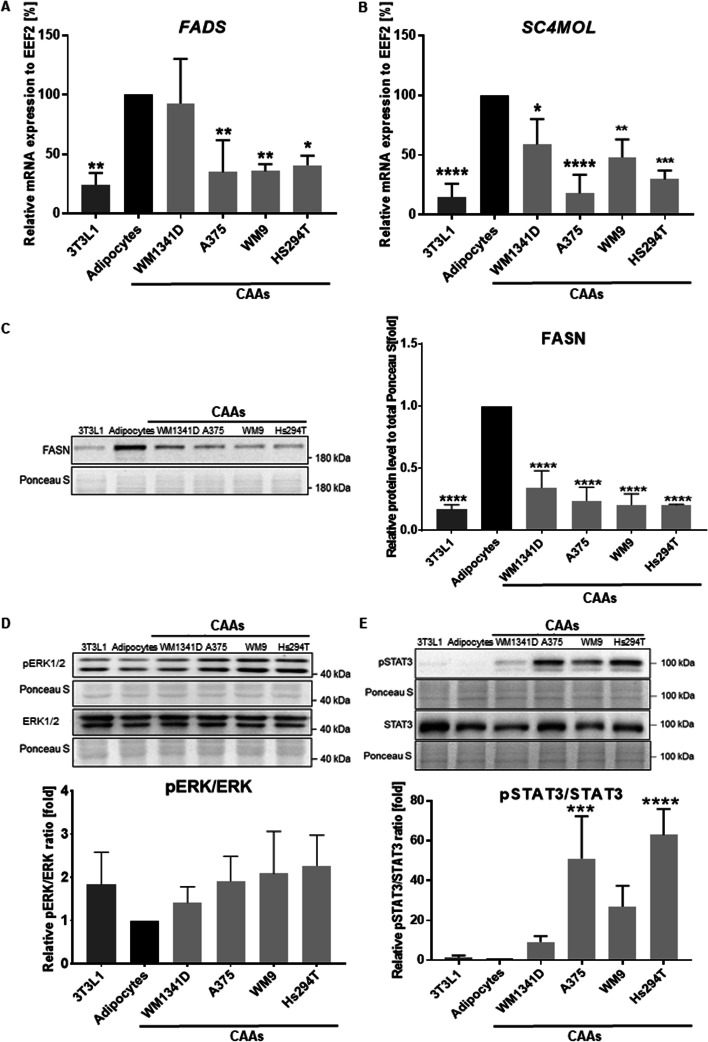
Analysis of mRNA expression and levels of proteins involved in lipid synthesis and lipolysis. The expression of FADS (**A**) and SC4MOL (**B**) was examined using RT–PCR. For quantification, the samples were normalized to the expression of EEF2. FASN level (**C**) and the pERK/ERK (**D**) and the pSTAT3/STAT3 (**E**) ratios were analyzed using western blotting. The signal was normalized to total protein content assessed by Ponceau S staining. Control constitutes non-treated adipocytes. The mean of at least three biological repetitions ± SD is shown. Asterisks indicate statistically significant differences between control adipocytes and 3T3L1/CAAs. The significance levels were set at *p* ≤ 0.05 (*), *p* ≤ 0.01 (**), *p* ≤ 0.001 (***), or *p* ≤ 0.0001 (****). *FADS* fatty acid desaturase, *SC4MOL* sterol-C4-methyl oxidase-like protein, *FASN* fatty acid synthase, *EEF2* eukaryotic translation elongation factor, *ERK* extracellular signal-regulated kinase, *STAT3* signal transducer and activator of transcription 3

To establish the cause of adipocyte delipidation, we evaluated the mRNA expression or protein levels of proteins involved in two processes that determine the amount of lipids in the cell: lipid synthesis and lipolysis. The expression of enzymes related to lipid synthesis [fatty acid desaturase (FADS), sterol-C4-methyl oxidase-like/methyl sterol monooxygenase 1 (SC4MOL), and fatty acid synthase (FASN)] was reduced in CAAs and 3T3L1 in comparison with control adipocytes (Fig. 5A–C). Mature fat cells have a functional FADS pathway [31]. SC4MOL catalyzes the oxidative decarboxylation of the C4 methyl groups and plays a central role in the cholesterol synthesis pathway, while FASN is regarded as a lipogenic protein [32, 33].

We also evaluated the activation status of the extracellular signal-regulated kinase (ERK) and signal transducer and activator of transcription 3 (STAT3) in examined cells. Two kinases, ERK1 and ERK2, are known collectively as ERK-1/2 or just ERK and fulfill analogous functions [34, 35]. ERK1/2 phosphorylates the β3-adrenergic receptor and hormone-sensitive lipase (HSL) and promotes the transcriptional downregulation of the adipose triglyceride lipase (ATGL) inhibitors, thus stimulating lipolysis [36]. Moreover, it was demonstrated that cytokines like IL-6 contribute to adipose wasting in patients with cancer through STAT3-dependent transcriptional changes that increase adipocyte lipolysis [37]. Western blot analysis of CAA lysates revealed a higher phosphorylation rate of ERK and pSTAT3 in CAAs compared with normal adipocytes (Fig. 5D, E). These differences were statistically significant in the case of STAT3 and adipocytes cocultured with A375 and Hs294T cells, but in the remaining cases, a strong upward trend was also visible. We also noticed that this increase was greater for cells cultured in the presence of more invasive melanoma cell lines (A375, WM9, and Hs294T) than for the less invasive one (WM1341D).

### Characterization of CAA secretome

Lipolysis can be stimulated by molecules secreted by adipocytes under the influence of cancer cells, including interleukin-6 [37], so we decided to examine the secretion pattern of molecules released by tested cells. We selected representative melanoma cell lines obtained from primary (WM1341D, A375) and metastatic (WM9) tumors for adipocyte activation and profiled the cytokines and angiogenesis factors in CAA-derived cell-conditioned media. These arrays were chosen because molecules secreted by adipocytes exert often proinflammatory and proangiogenic effects on other cells [38].

**Fig. 6:**
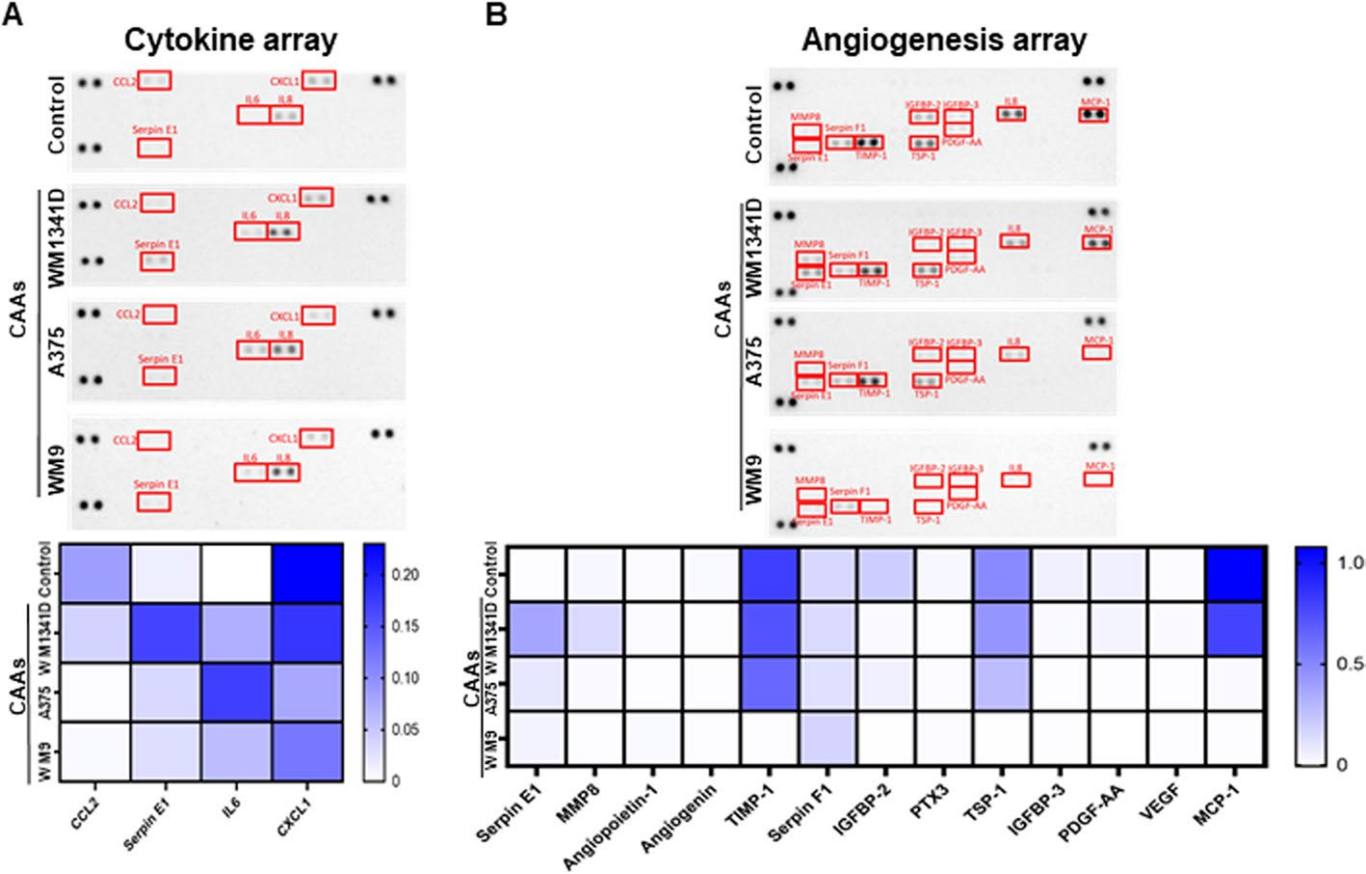
CAA secretome analysis. To identify proteins secreted by control adipocytes and CAAs cocultured with melanoma cells (lines: WM1341D, A375, WM9) present on Transwell inserts, conditioned media were analyzed using cytokines (**A**) and angiogenesis (**B**) arrays. Based on obtained signals, quantitative analysis was performed. Densitometric data were normalized to reference spots and are presented in the form of heatmaps, where darker blue indicates a higher increase in signal. *CCL2 (MCP-1)* C–C motif chemokine ligand 2, *IL6* interleukin 6, *CXCL1* C-X-C motif chemokine ligand 1, *PTX3* pentraxin 3, *IGFBP-2* insulin-like growth factor binding protein 2, *IGFBP-3* insulin-like growth factor binding protein 3, *VEGF* vascular endothelial growth factor, *PDGF-AA* platelet-derived growth factor AA, *TIMP-1* tissue inhibitor of metalloproteinases 1, *TSP-1* thrombospondin 1, *MMP8* matrix metalloprotease 8

Figure 6 shows secreted proteins with expression that differs between control adipocytes and CAAs. In the case of cytokines, SerpinE1 and IL-6 secretion was increased in CAAs, while secretion of CCL2 and CXCL1 was reduced in CAAs in comparison with control adipocytes (Fig. 6A). The angiogenesis array allowed us to detect the decrease in the level of most secreted angiogenic mediators in CAAs following melanoma coculture except for SerpinE1. High reductions were noticed in the case of tissue inhibitor of metalloproteinase 1 (TIMP-1), thrombospondin 1 (TSP-1), and CCL2 (MCP-1) (Fig. 6B).

### Metabolic changes in CAAs

**Fig. 7:**
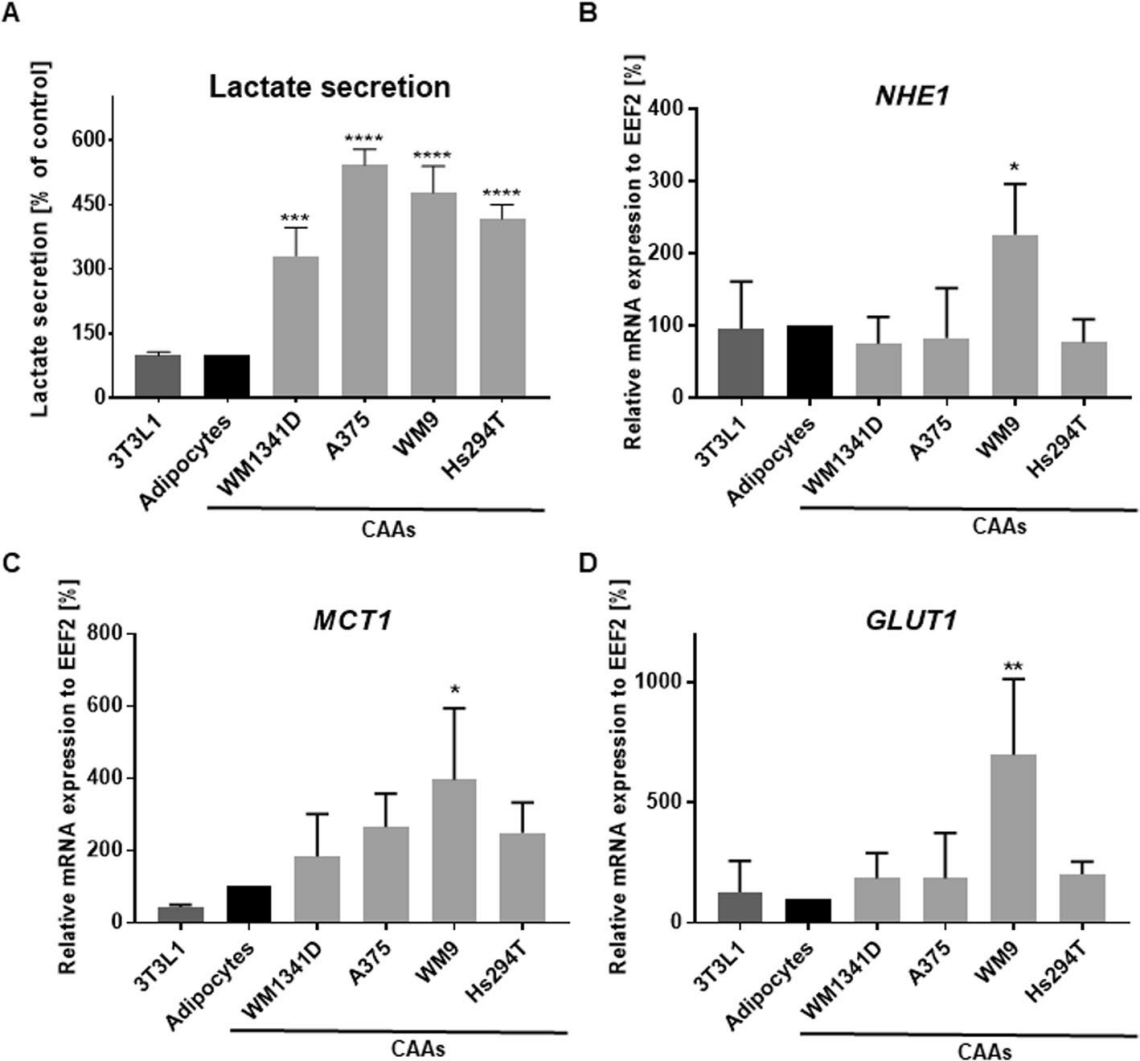
Influence of melanoma cells on lactate secretion (**A**), ions (**B**, **C**), and glucose transport (**D**) by 3T3L1 cells, control adipocytes, and CAAs. CAAs were obtained from adipocytes cultured with melanoma cells (lines: WM1341D, A375, WM9, and Hs294T) present on Transwell inserts. **A** The level of secreted lactate was measured using a chemiluminescent reaction in a cell-conditioned medium devoid of serum, in which cells were grown for 24 h. The expression of NHE1 (**B**), MCT1 (**C**), and GLUT1 (**D**) was estimated through real-time PCR analysis. For quantification, the samples were normalized against the expression of EEF2. The mean of at least three independent repetitions ± SD is shown. Asterisks indicate statistically significant differences between control cells and 3T3L1/CAAs. The significance levels were set at *p* ≤ 0.05 (*), *p* ≤ 0.01 (**), *p* ≤ 0.001 (***), or *p* ≤ 0.0001 (****). *NHE1* sodium/hydrogen exchanger 1, *MCT1* monocarboxylate transporter 1, *GLUT1* glucose transporter 1, *EEF2* eukaryotic translation elongation factor 2

Extracellular acidification can be induced by lactate secretion and is a relevant factor that modifies the TME and affects cancer progression. Hence, we evaluated the level of lactate released by 3T3L1 fibroblasts, control adipocytes, and CAAs. Extensive secretion of this metabolite was observed in adipocytes after coculture with melanoma cells (Fig. 7A). Moreover, we performed RT–PCR analysis and found higher expression of ion transporters in CAAs compared with control cells: Na^+^/H^+^ exchanger (NHE1) and monocarboxylate transporter 1 (MCT1). However, the only significant difference was in the case of adipocytes treated with metastatic WM9 melanoma cells (Fig. 7B, C).

NHE1 is strongly involved in the maintenance of intracellular pH homeostasis, while lactate is extruded by monocarboxylate transporter MCT1, which transports lactate together with H^+^ ions in the same direction [39]. The expression of glucose transporter isoform 1 (GLUT1) was increased in adipocytes treated with melanoma, but again, the only statistically significant difference was in the case of WM9 cells (Fig. 7D).

### Changes in melanoma cells proliferation under the influence of adipocytes

**Fig. 8:**
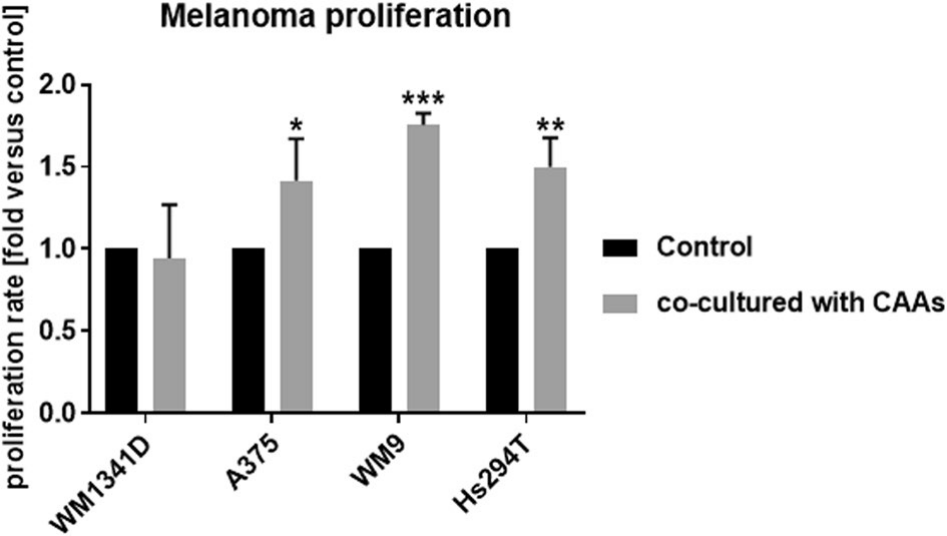
Effect of coculture with adipocytes on the proliferation of melanoma cells. Melanoma cells (lines: WM1341D, A375, WM9, and Hs294T) were cocultured with adipocytes present on Transwell inserts for 7 days. Next, melanoma cells were seeded on a 96-well plate, and their proliferation rate was calculated as a ratio of the spectrophotometric signal after 48 h to the spectrophotometric signal at T0. Results are expressed as the mean (fold change) ± SD of three independent experiments. Asterisks above the bars express significance vs. control. The significance levels were set at *p* ≤ 0.05 (*), *p* ≤ 0.01 (**), and *p* ≤ 0.001 (***)

The changes that we detected in CAAs, such as delipidation and increased lactate secretion, may affect the proliferation of tumor cells. Therefore, we assessed how adipocytes affect melanoma cell proliferation. The results demonstrated that three of four melanoma cell lines (the more invasive ones) proliferated faster under the influence of CAAs (Fig. 8). We hypothesized that nutrients released by adipocytes may support the proliferation of melanoma cells [19, 28, 40].

## Discussion

Traditionally, adipocytes have been described as mainly reservoirs of nutrients, but now we know that they can also play a secretory role, and thus affect the functioning of other cells and tissues. Subsequently, it was shown that in many types of cancer, obesity contributes to a poorer prognosis for patients. This relationship was particularly noticeable in the case of breast and colon cancer, but such a relationship was also found in melanoma [3, 41, 42]. Further studies have shown that adipocytes can support cancer progression at many levels, mainly by secreting adipokines that stimulate the proliferation and migration of cancer cells, but also by providing nutrients [43, 44].

Research in this area has usually focused on determining the changes occurring in cancer cells under the influence of adipocytes, and much less attention has been paid to the influence of cancer cells on adipocytes. This topic has been described to a higher extent for breast cancer, but data for other cancers, including melanoma, are very limited. We decided to address this issue because aggressive melanoma cells can migrate to the adipocyte-rich hypodermic layer of the skin and strongly impact the biology and functions of fat cells.

It was demonstrated that CAAs surrounding breast cancer are smaller and exhibit lower lipid content, reduced levels of adipocyte markers (leptin, adiponectin, resistin, FABP4), and overexpression of proinflammatory cytokines and matrix-remodeling proteins [43–46]. To establish the influence of melanoma cells on adipocytes in their vicinity, we focused on similar effects. The feature characterizing the transformation of adipocytes into CAAs is the inhibition of adipogenesis: the process of preadipocytes’ conversion into mature adipocytes. In the terminal stage of this transformation, several adipocyte-specific genes are induced: FABP4, leptin, resistin, adiponectin, and perilipin [28, 47].

We noticed that mature adipocytes express a high level of selected adipokines (leptin, adiponectin, resistin), lipid transport protein (FABP4), and enzymes related to lipid synthesis (FADS, SC4MOL, FASN), while their levels decrease in adipocytes cocultured with melanoma cells (Figs. 2, 5). Leptin induces intracellular signaling in adipocytes promoting adipogenesis [48], so its lowered expression in CAAs could be expected. A reduced level of adiponectin in adipocytes cocultured with melanoma cells was also observed by Zoico et al. [40]. This adipokine regulates glucose and lipid metabolism and can inhibit cell growth and induce apoptosis in cancer cells, mainly through activation of AMPK, MAPK, PI3K/AKT, or STAT3 signaling [49].

Mouse melanoma tumors presented a markedly higher growth rate in adiponectin-deficient mice [3]. Adipocyte protein 2 (FABP4) is expressed in fat cells, where it plays a role as a transporter of fatty acids that facilitates their entry into the plasma membrane, helps in their intracellular transport, and affects lipid and glucose metabolism by increasing fatty acid levels [50]. Fatty acid synthase (FASN) is engaged in the final steps of the de novo biogenesis of fatty acids and in adipocyte maturation [51, 52]. Fatty acid desaturase-1 (FADS1) is an enzyme that participates in the biosynthesis of long-chain polyunsaturated fatty acids eicosapentaenoic and arachidonic acid, which are essential elements of cellular membranes and also play a role in cell signaling, inflammation, and death [53].

SC4MOL catalyzes two sequential steps that are essential to convert squalene to cholesterol [32]. To the best of our knowledge, this is the first study showing the occurrence of downregulation of these enzymes’ expression in melanoma-associated adipocytes. Decreased expression levels of molecules that are characteristic of differentiated adipocytes confirmed that all of the examined melanoma cell lines induced transformation of adipocytes into CAAs.

We also noticed that adipocytes cocultured with melanoma cells change their morphology. They changed from rounded cells to spindle-shaped and showed morphology similar to that of fibroblasts with a well-organized actin cytoskeleton. In addition, CAAs showed a gelatin digestion pattern that was more similar to that of 3T3L1 fibroblasts than to that of mature adipocytes (Fig. 3A). Zoico et al. also observed the appearance of dedifferentiated cells with a fibroblast-like phenotype after coculture of adipocytes with melanoma. Moreover, it was shown that adipocytes from the breast cancer tumor niche can be reprogrammed into CAAs or can dedifferentiate into fibroblast-like cells, which are also called adipocyte-derived fibroblasts [54]. This phenomenon can occur because adipocytes present high plasticity, and even mature cells may dedifferentiate to multipotent cells [55, 56].

This phenotypic transition from adipocytes to fibroblast-like cells has mainly been demonstrated for breast cancer, and very limited data are available in the literature regarding other types of cancer [54, 57–60]. In breast cancer, the appearance of adipocyte-derived fibroblasts is stimulated through the activation of the Wnt/β-catenin pathway in response to Wnt3a secreted by the tumor [54, 61]. In the CAAs that we cocultured with melanoma, the β-catenin level was slightly elevated in comparison with control cells, but this difference was not statistically significant (data not shown).

Due to the detection of fibroblast features in the obtained CAAs, we determined the level of proteins that may act as markers of these cells, such as vimentin and TGFβRIII. We found that the TGFβRIII level was elevated in CAAs cultured with Hs294T and WM9 cells, while the vimentin level was significantly increased in CAAs cocultured with Hs294T cells. TGFβRIII is expressed in fibroblasts, where it takes part in the activation of type II TGF-β receptor and initiation of the TGF-β-induced signal transduction pathway [62].

Vimentin is highly expressed in fibroblasts and is recognized as a marker of epithelial–mesenchymal transition [63, 64], so its elevation in the examined cells is not surprising. Interestingly, we also detected this protein in mature adipocytes, which may be due to the fact that vimentin forms a scaffold around the lipid droplets in adipocytes and participates in lipolysis through direct hormonally regulated interactions with hormone sensitive lipase [65, 66]. Despite this, we found out that melanoma-associated adipocytes exhibit features of fibroblast-like cells rather than morphology typical of adipocytes.

Previous reports show that adipocytes cocultured with breast cancer cells have a reduced number and size of lipid droplets, which correlate with a significant decrease in lipid accumulation [43]. Therefore, we determined its level in the CAAs. Qualitative and quantitative analysis indicated that the number of lipid droplets and the amount of accumulated lipids decreased in CAAs in comparison to control cells. Lipid droplets fill most of the adipocyte volume, and the fat consists predominantly of triglycerides [67]. The level of perilipin, which decorates lipid droplets and protects them from the access of lipases [68], was also reduced. This result may at least partially explain the downregulated lipid content in achieved CAAs.

Wagner et al. showed that after melanoma injection into subcutaneous adipose tissue of a mouse, adipocytes present in the tumor vicinity demonstrated a decreased lipid content related to increased lipolysis [69]. In an ovarian cancer model, fatty acids released by CAAs are transferred to cancer cells to support tumor growth by fueling fatty acid oxidation (FAO) [16]. Wen et al. indicated that the level of glycerol released by adipocytes to the culture medium was elevated after incubation with colorectal cancer cells, suggesting that cancer cells stimulate lipolysis [70]. Pancreatic cancer cells also promote lipolysis in subcutaneous adipocytes through a mechanism based on exosomal adrenomedullin [61, 71].

The process of lipolysis also participates in cachexia, a syndrome that appears in patients with cancer and is associated with the loss of adipose and muscle mass. Das et al. showed that the growth of melanoma tumors in mice led to upregulation of lipolysis, a decrease of fat mass, and the loss of skeletal muscle volume, independent of food intake. In cachexia, adipocytes exhibit a reduced level of adipogenic transcription factors and differentiation markers and an increased level of lipolytic enzymes, such as hormone-sensitive lipase (HSL) or adipose triglyceride lipase (ATGL), which is not found in CAAs [67, 72].

We have not observed upregulation of mRNA encoding HSL in melanoma-associated adipocytes as well (data not shown), which supports the idea that the examined cells are CAAs. Nevertheless, the observed CAA delipidation may be related to reduced expression of genes encoding enzymes involved in fat synthesis in these cells (Fig. 5). As mentioned, we noticed a lower level of mRNA of SC4MOL (implicated in cholesterol biosynthesis), FADS1, and FASN in adipocytes cocultured with melanoma cells. Therefore, a significant decrease in the number of lipid droplets may be connected not only with the downregulation of perilipin, which protects lipid droplets, but also with alteration in the expression of enzymes involved in de novo lipid synthesis.

To understand the basis of lipolysis in CAAs, we determined the level of activation of signaling pathways that may be associated with this process. We observed a trend of increasing pERK/ERK ratio and a statistically significant increase in the pSTAT/STAT ratio in CAAs relative to mature adipocytes. The MAPK pathway is involved in the regulation of adipogenesis and lipolysis. It was previously shown that activation of ERK may lead to a reduction of the level of perilipin [73], which protects lipid droplets against lipolysis. Moreover, it was demonstrated that the IL-6 family of cytokines required JAK/STAT3-dependent transcriptional changes to induce adipocyte lipolysis during cachexia [37, 74]. We hypothesized that a mechanism based on increased IL-6 and pSTAT3 levels could be responsible for the stimulation of lipolysis in the CAAs.

Due to this, as well as the fact that adipocytes secrete many factors affecting the cells present in the TME, we examined the secretion pattern of proteins in the tested cells. We detected increased secretion of SerpinE1 and IL-6 by CAAs and decreased amounts of CCL2, CXCL1, TIMP-1, and TSP-1 in the media collected from these cells in comparison to control adipocytes’ media (Fig. 6). Elevated levels of IL-6 and SerpinE1 were also demonstrated in the case of CAAs related to breast cancer [43]. IL-6 is often released by CAAs and is a relevant signaling molecule that affects the immune system, lipid metabolism, insulin resistance, and mitochondrial activity. As mentioned, it may also activate pSTAT3. Moreover, it promotes melanoma progression [28, 61, 75].

Plasminogen activator inhibitor-1 (PAI-1), also known as SerpinE1, is expressed in a majority of tissues, including adipocytes of subcutaneous and visceral white adipose tissue [42, 76]. This molecule is involved in glucose and lipid metabolism [77]. PAI-1 can increase metastasis of cancer cells because it is involved in the activation of plasminogen from a zymogen to an active protease, which then contributes to ECM remodeling by activating MMPs [42]. Moreover, PAI-1 inhibits the differentiation of adipocytes by decreasing the expression of PPARγ, C/EBPα, and FABP4 [78]. We also observed reduced expression of the latter protein in CAAs (Fig. 2).

We also detected a reduced level of TIMP-1. The overexpression of TIMP-1 directly inhibits the tumorigenic and metastatic potential of B16-F10 cells in mice [79, 80]. This may suggest that a decreased amount of this protein secreted by CAAs (in comparison with control adipocytes) may support the invasion of melanoma cells in their vicinity. TSP1 in obesity induces inflammation and promotes weight gain and metabolic dysfunction. It also exerts proliferative effects on adipocytes and may affect adipocyte fatty acid uptake [81, 82]. Furthermore, adipocytes from obese individuals present significantly higher levels of CCL2, while adipogenesis is also regulated by CXCL1 [83, 84].

As mentioned, adipocytes may release fatty acids, which provide nutrients to the cancer cells. However, apart from lipids, the other molecules secreted by adipocytes may also be a source of energy for cancer cells. The transport of lactate into the TME by the MCT1 can be coupled to the cotransport of H^+^ ions and cause acidification of the microenvironment [85]. Moreover, NHE1, a reversible antiporter, also expels H^+^ ions generated in the cytosol to the tumor niche, which reduces pH inside the cell [39]. Nevertheless, it was shown that lactate secreted by stromal cells can become an energy source for cancer cells [86].

It was previously demonstrated that breast cancer cells can promote catabolic processes in adipocytes, leading to the release of high‑energy metabolites like lactic acid, pyruvic acid, FFA, and ketone bodies [28, 41]. Cancer cells can then consume these metabolites. We have shown that CAAs secrete an increased amount of lactate (Fig. 7A). Moreover, in comparison with control cells, elevated levels of transporters of glucose (GLUT1), lactate (MCT1), and H^+^ ions (NHE1) were observed in CAAs, and in the case of CAAs cultured with WM9 cells the result was statistically significant (Fig. 7B–D). These results were indirectly confirmed with data obtained by Zoico et al. They showed that after coculture of 3T3L1-derived adipocytes with melanoma cells, the pH of the medium was significantly reduced when compared to the control medium of adipocytes grown alone [40], which may be related to lactate secretion.

We hypothesize that melanoma cells may support the intake of glucose through GLUT1 present on adipocytes, which then metabolize this carbohydrate into lactate and secrete it via MCT1 into the medium. Both released lactate and fatty acids can provide an energy source for melanoma cells and support their proliferation. To confirm this, we performed a melanoma cell proliferation assay after their coculture with adipocytes and noted that CAA-treated A375, WM9, and Hs294T cells exhibited significantly higher proliferation rates than control melanoma cells. This may be related to the fact that FAs released from adipocytes constitute nutrients and thus support cancer cell growth.

**Fig. 9:**
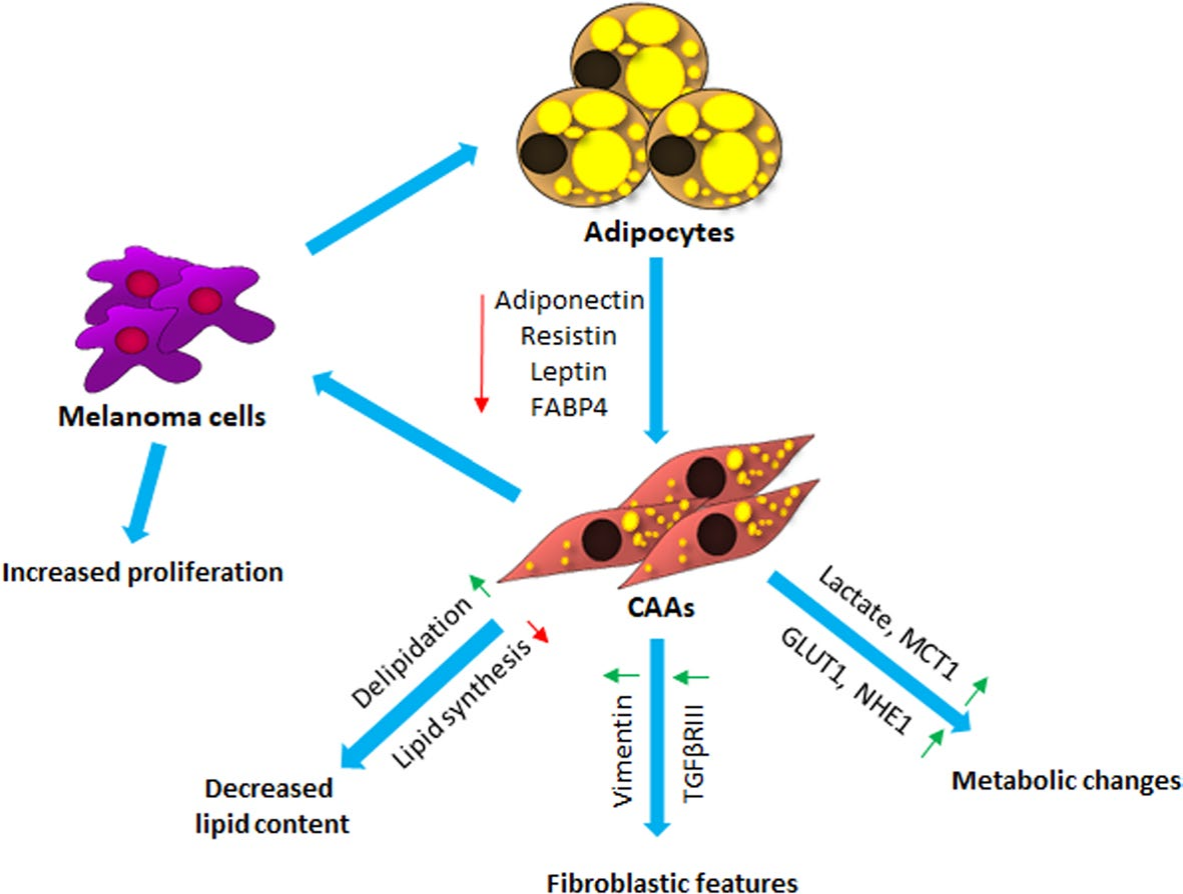
Crosstalk between melanoma cells and CAAs. *CAAs* cancer-associated adipocytes, *FABP4* fatty acid binding protein 4, *TGFβRIII* TGFβ receptor type III, *MCT1* monocarboxylate transporter 1, *NHE1* sodium–hydrogen antiporter 1, *GLUT1* glucose transporter 1. Green arrows show upregulation, and red arrows show reduction

In summary, we found that melanoma-associated adipocytes exhibit reduced levels of adipogenesis markers, in parallel with the dedifferentiation of adipocytes into fibroblast-like cells. Simultaneously, adipocytes reprogrammed by cancer cells may support melanoma cells by secretion of lactate and fatty acids, which can act as an energy source for melanoma cells and support their proliferation (Fig. 9). Our research demonstrated that through the transformation of adipocytes, melanoma cells induce profound metabolic changes in the TME, which could be a potential therapeutic target to reduce the invasiveness of cancer cells. In the future, we are considering conducting extensive transcriptome studies on CAAs isolated from biopsies derived from patients with melanoma, which would allow us to perform a deeper analysis of the data described here.

## Conclusions

We found that melanoma-associated adipocytes exhibit reduced levels of adipogenesis markers and a significant decrease in the number of lipid droplets in parallel with the dedifferentiation of adipocytes into fibroblast-like cells (Fig. 9). Simultaneously, with the appearance of fibroblast-like cells in coculture, we observed a significantly increased release of lactate and expression of the transporters of glucose, lactate, and H^+^ ions, which probably reflects a profound metabolic change in the TME. Both secreted lactate and fatty acids can provide an energy source for melanoma cells and support their proliferation.

## Supplementary Information

**Additional file 1:** The nitroceluluse membranes on which are visualized bands corresponding to selected proteins (ERK1/2, pERK1/2, FASN, STAT3, pSTAT3, TGFßRIII, vimentin) or Ponceau S staining.

## Data Availability

The datasets used and/or analyzed during the current study are available from the corresponding author on reasonable request.

## Abbreviations

CAA: Cancer-associated adipocytes
TME: Tumor microenvironment
FFA: Free fatty acids
IL-6: Interleukin 6
IL-8: Interleukin 8
CCL2: C–C motif chemokine ligand 2
CXCL1: C-X-C motif chemokine ligand 1
PTX3: Pentraxin-related protein
PAI-1: Plasminogen activator inhibitor-1
IGFBP: Insulin-like growth factor-binding protein
VEGF: Vascular endothelial growth factor
IGF-I: Insulin-like growth factor 1
PI3K: Phosphoinositide 3-kinase
AKT: Protein kinase B
MAPK: Mitogen-activated protein kinase
JAK: Janus kinase
STAT: Signal transducer and activator of transcription
FASN: Fatty acid synthase
TGFβRIII: TGFβ receptor III
TIMP-1: Tissue inhibitor of metalloproteinase 1
TSP-1: Thrombospondin 1
MMP8: Matrix metalloprotease 8
PDGF-AA: Platelet-derived growth factor AA
EEF2: Eukaryotic translation elongation factor 2
FABP4: Fatty acid binding protein
SC4MOL: Sterol-C4-methyl oxidase-like protein
PLIN1: Perilipin 1
FADS1: Fatty acid desaturase 1
MCT1: Monocarboxylate transporter 1
NHE1: Sodium–hydrogen antiporter 1
GLUT1: Glucose transporter 1
