# Supplementary material for: Melanoma cells induce dedifferentiation and metabolic changes in adipocytes present in the tumor niche
Source: Cell Mol Biol Lett. 2023 Jul 22;28:58. doi: 10.1186/s11658-023-00476-3 (PMC10363323; doi:10.1186/s11658-023-00476-3)

ERK 1/2 Adipocytes additional control – adipocytes cultured in medium dedicated only for adipocytes (only with BCS)

ERK 1/2 Repetition 1 Ponceau staining

3T3L1 Adipocytes additional ctrl Adipocytes ctrl WM1341D CAA A375 CAA WM9 CAA Hs294T CAA

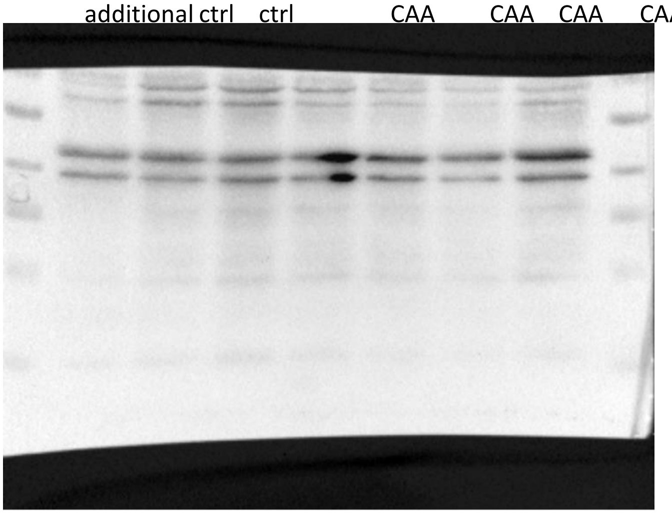

3T3L1 Adipocytes additional ctrl Adipocytes ctrl WM1341D CAA A375 CAA WM9 CAA Hs294T CAA

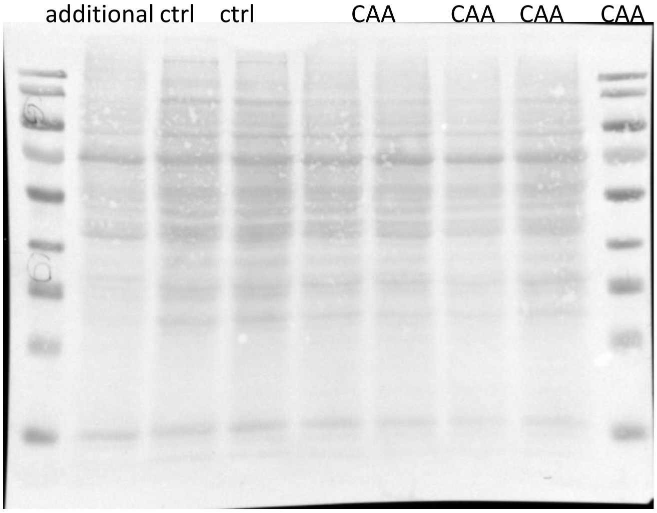

ERK 1/2 Repetition 2 Ponceau staining

3T3L1 Adipocytes additional ctrl Adipocytes ctrl WM1341D CAA A375 CAA WM9 CAA Hs294T CAA

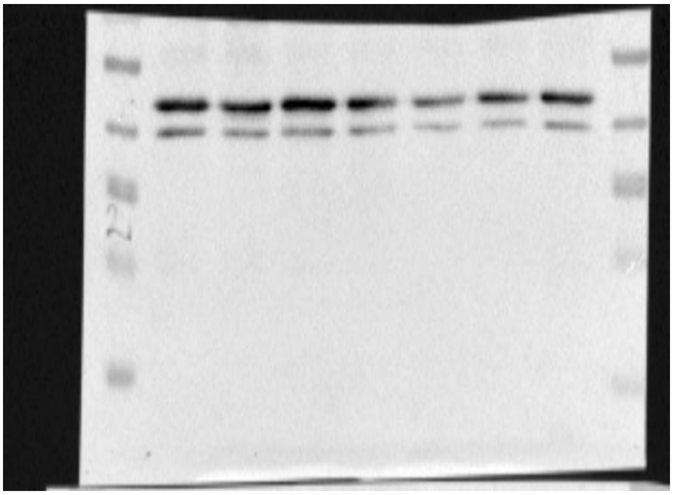

3T3L1 Adipocytes additional ctrl Adipocytes ctrl WM1341D CAA A375 CAA WM9 CAA Hs294T CAA

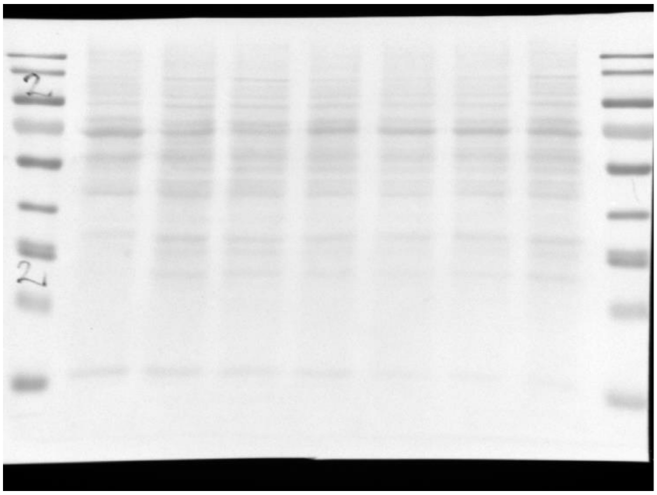

ERK 1/2 Repetition 3 Ponceau staining

3T3L1 Adipocytes WM1341D CAA A375 CAA WM9 CAA Hs294T CAA

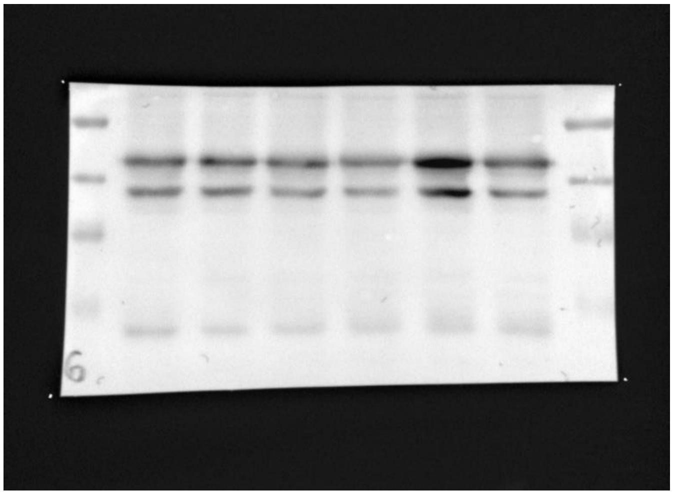

3T3L1 Adipocytes WM1341D CAA A375 CAA WM9 CAA Hs294T CAA

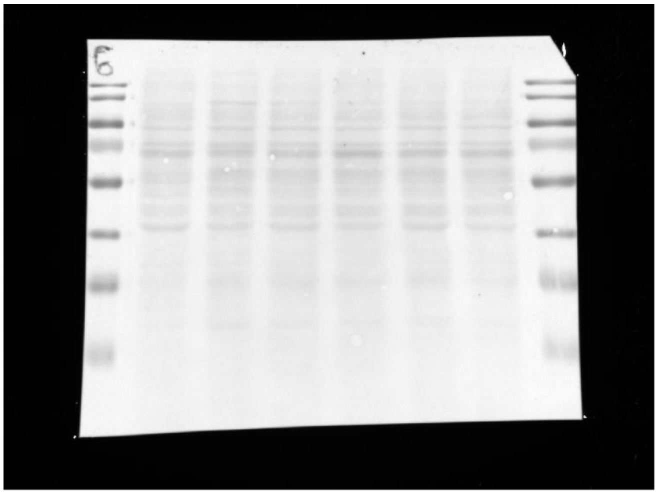

pERK 1/2

Adipocytes additional control – adipocytes cultured in medium dedicated only for adipocytes (only with BCS)

pERK 1/2 — Repetition 1 — Ponceau staining

3T3L1 | Adipocytes additional ctrl | Adipocytes ctrl | WM1341D CAA | A375 CAA | WM9 CAA | Hs294T CAA

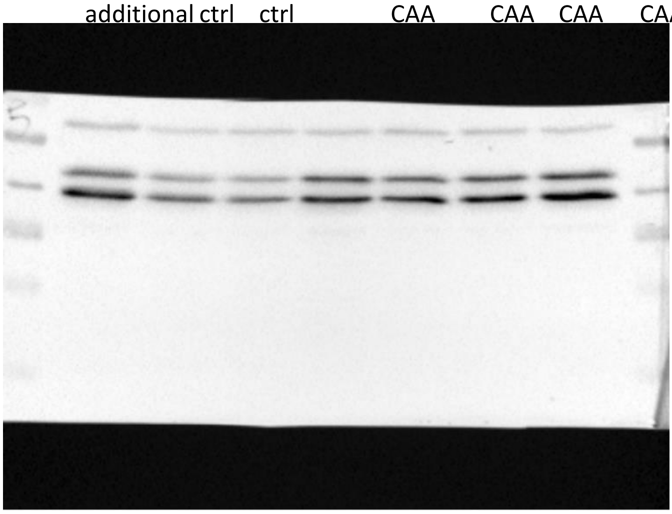

3T3L1 | Adipocytes additional ctrl | Adipocytes ctrl | WM1341D CAA | A375 CAA | WM9 CAA | Hs294T CAA

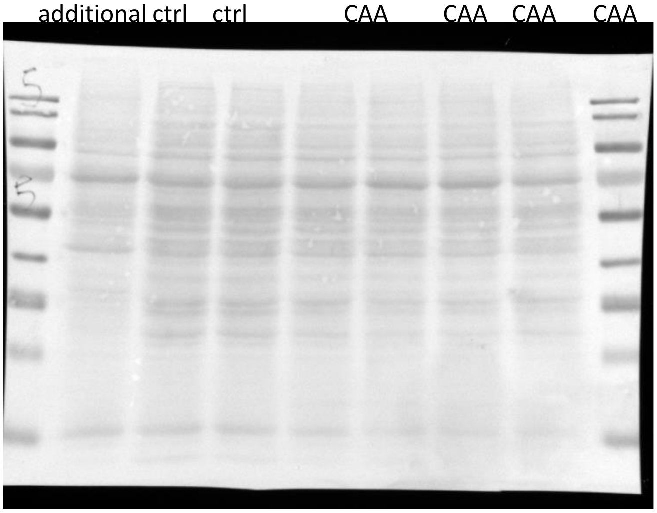

pERK 1/2 — Repetition 2 — Ponceau staining

3T3L1 | Adipocytes additional ctrl | Adipocytes ctrl | WM1341D CAA | A375 CAA | WM9 CAA | Hs294T CAA

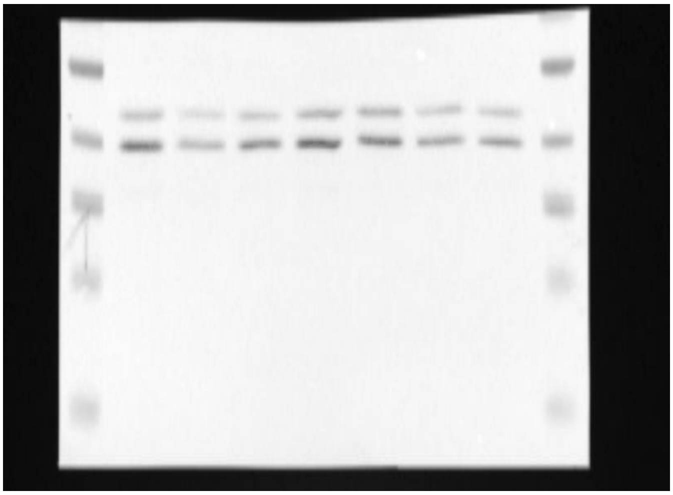

3T3L1 | Adipocytes additional ctrl | Adipocytes ctrl | WM1341D CAA | A375 CAA | WM9 CAA | Hs294T CAA

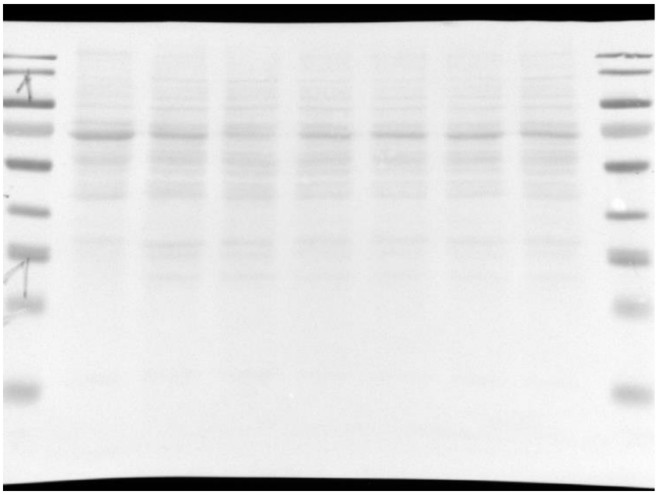

pERK 1/2 — Repetition 3 — Ponceau staining

3T3L1 | Adipocytes | WM1341D CAA | A375 CAA | WM9 CAA | Hs294T CAA

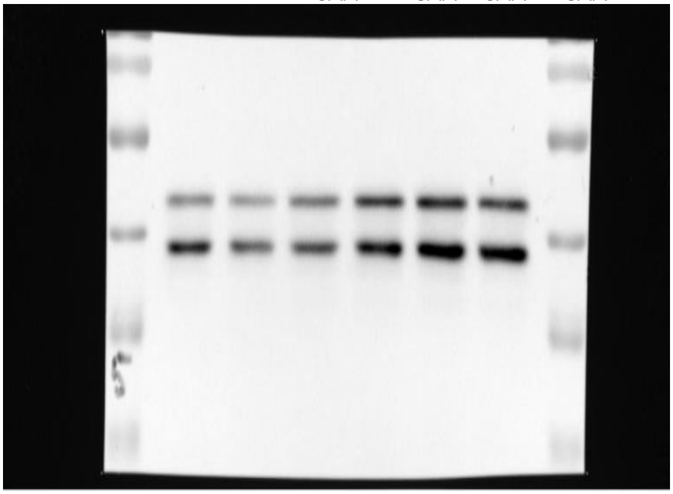

3T3L1 | Adipocytes | WM1341D CAA | A375 CAA | WM9 CAA | Hs294T CAA

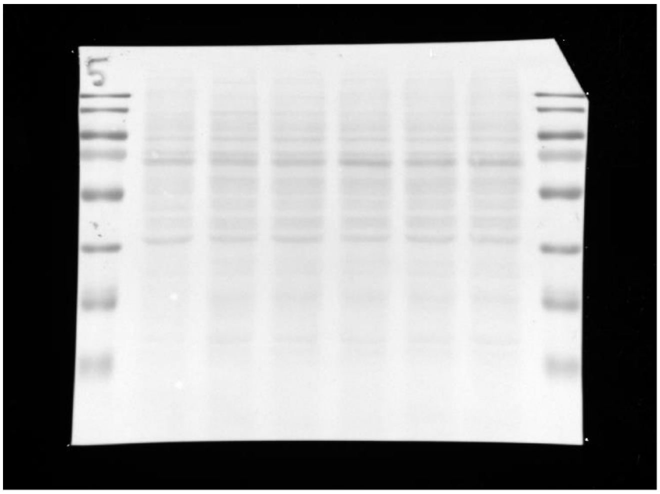

FASN

Repetition 1

FASN

3T3L1 Adipocytes WM1341D A375 WM9 Hs294T
CAA CAA CAA CAA

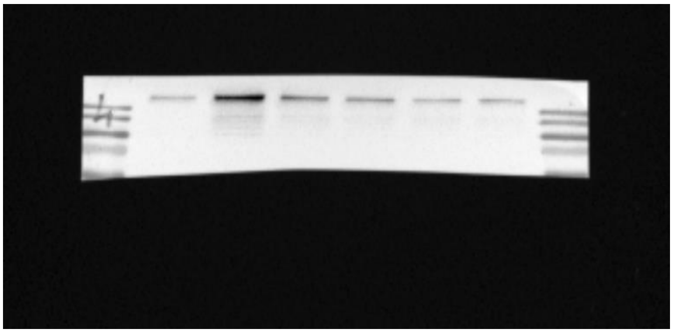

Ponceau staining

3T3L1 Adipocytes WM1341D A375 WM9 Hs294T
CAA CAA CAA CAA

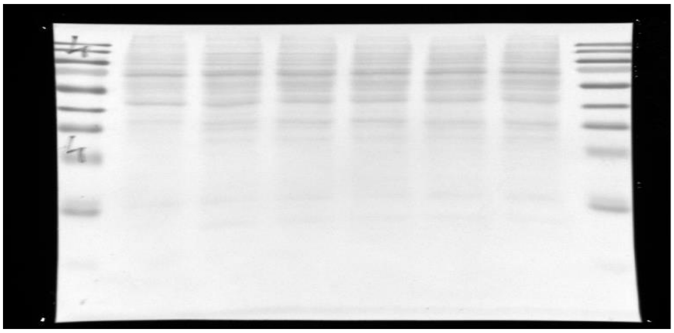

Repetition 2

FASN

3T3L1 Adipocytes WM1341D A375 WM9 Hs294T
CAA CAA CAA CAA

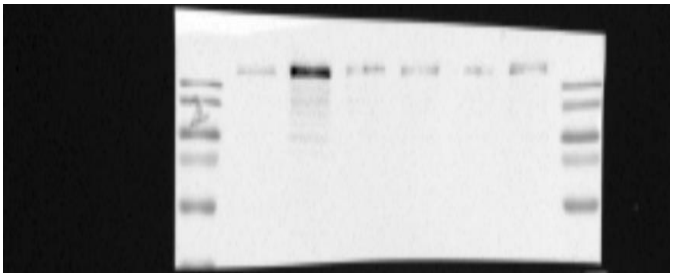

Ponceau staining

3T3L1 Adipocytes WM1341D A375 WM9 Hs294T
CAA CAA CAA CAA

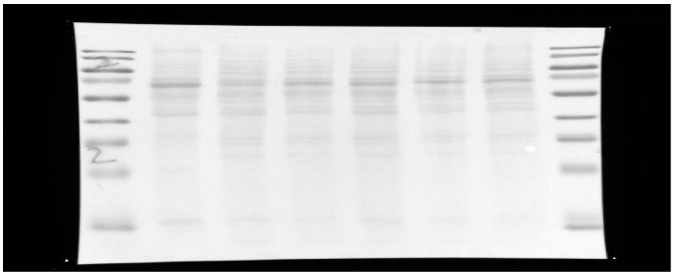

Repetition 3

FASN

3T3L1 Adipocytes WM1341D A375 WM9 Hs294T
CAA CAA CAA CAA

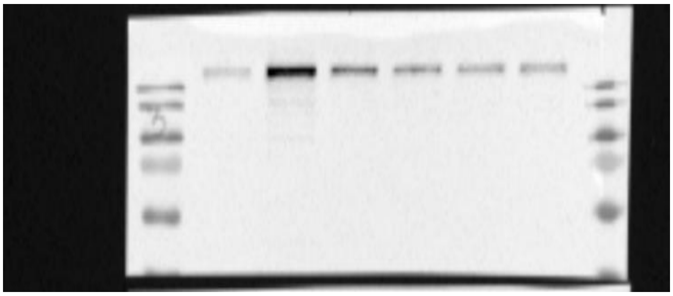

Ponceau staining

3T3L1 Adipocytes WM1341D A375 WM9 Hs294T
CAA CAA CAA CAA

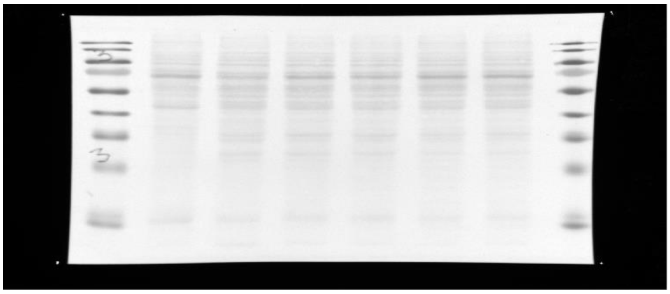

Repetition 4

FASN

3T3L1 Adipocytes WM1341D A375 WM9 Hs294T
CAA CAA CAA CAA

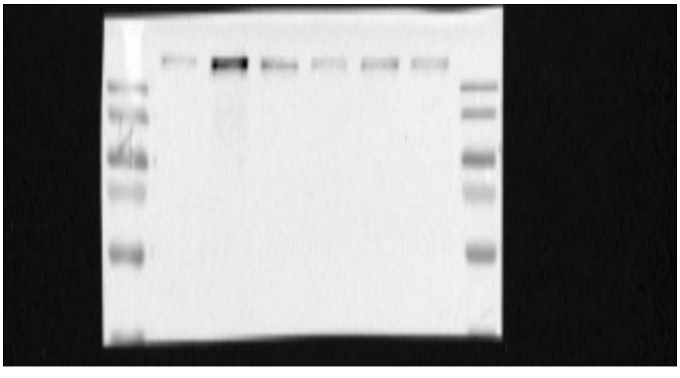

Ponceau staining

3T3L1 Adipocytes WM1341D A375 WM9 Hs294T
CAA CAA CAA CAA

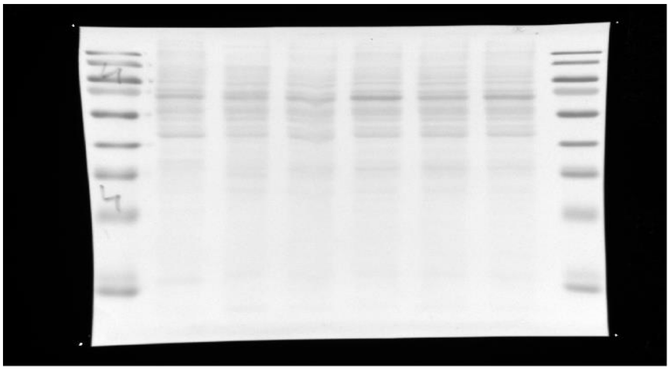

STAT3

Repetition 1

STAT3

3T3L1 Adipocytes WM1341D A375 WM9 Hs294T
CAA CAA CAA CAA

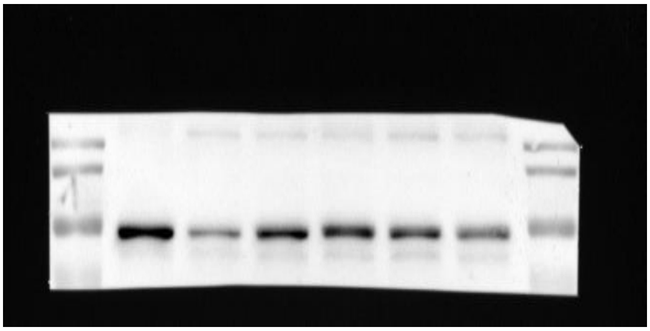

Ponceau staining

3T3L1 Adipocytes WM1341D A375 WM9 Hs294T
CAA CAA CAA CAA

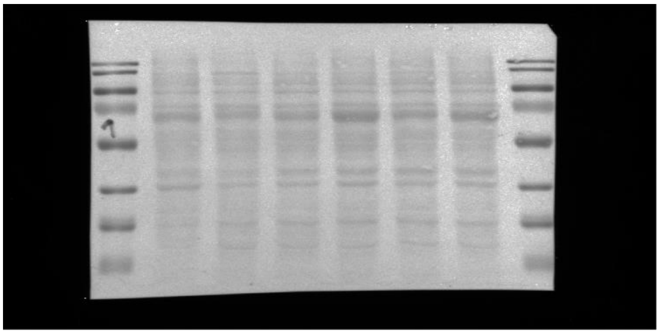

Repetition 2

STAT3

3T3L1 Adipocytes WM1341D A375 WM9 Hs294T
CAA CAA CAA CAA

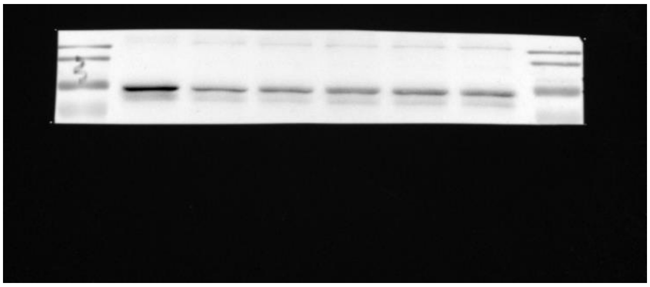

Ponceau staining

3T3L1 Adipocytes WM1341D A375 WM9 Hs294T
CAA CAA CAA CAA

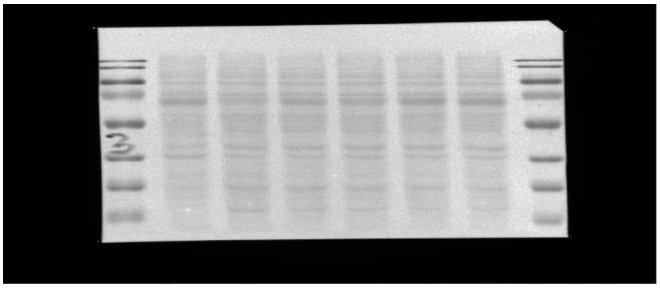

Repetition 3

STAT3

3T3L1 Adipocytes WM1341D A375 WM9 Hs294T
CAA CAA CAA CAA

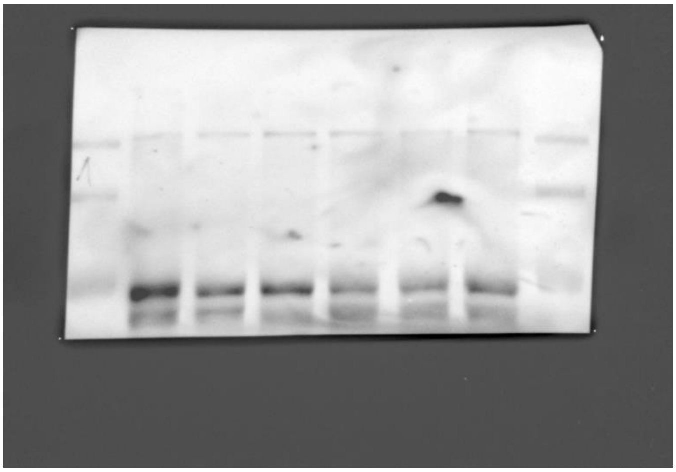

Ponceau staining

3T3L1 Adipocytes WM1341D A375 WM9 Hs294T
CAA CAA CAA CAA

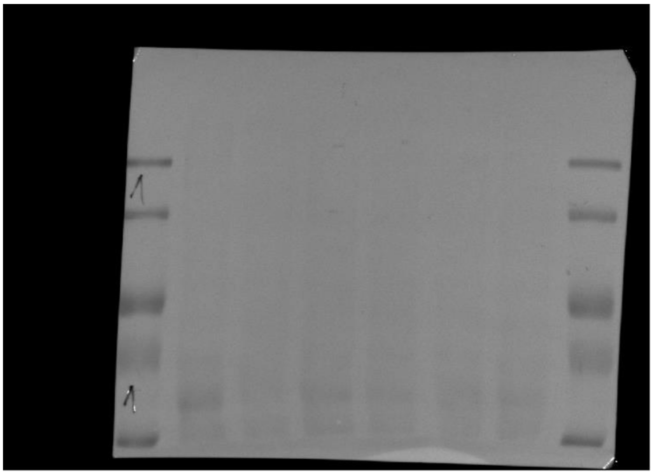

pSTAT3

pSTAT3 — Repetition 1 — Ponceau staining

3T3L1 Adipocytes WM1341D CAA A375 CAA WM9 CAA Hs294T CAA

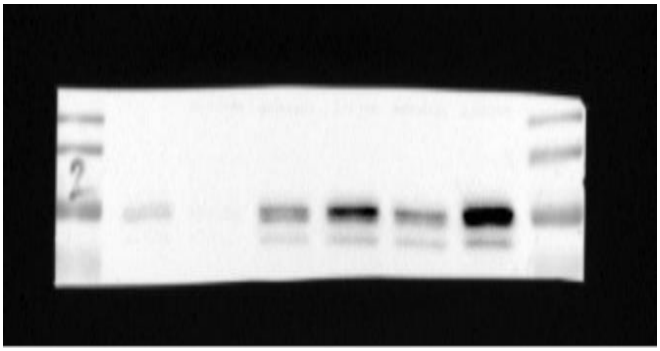

3T3L1 Adipocytes WM1341D CAA A375 CAA WM9 CAA Hs294T CAA

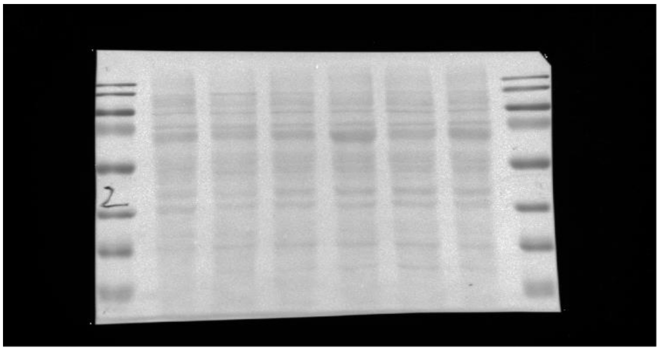

pSTAT3 — Repetition 2 — Ponceau staining

3T3L1 Adipocytes WM1341D CAA A375 CAA WM9 CAA Hs294T CAA

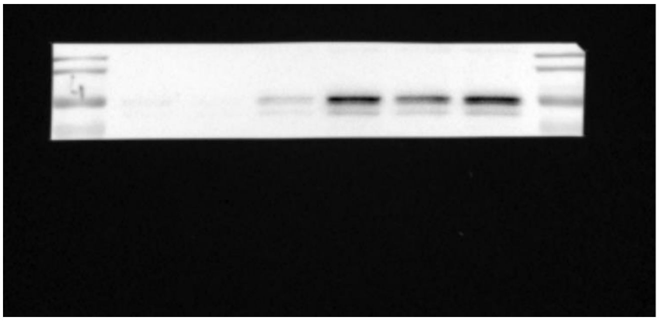

3T3L1 Adipocytes WM1341D CAA A375 CAA WM9 CAA Hs294T CAA

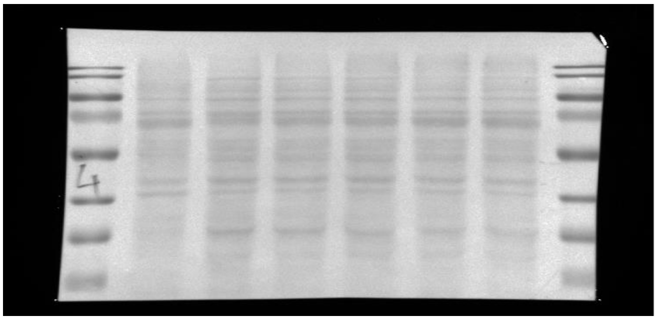

pSTAT3 — Repetition 3 — Ponceau staining

3T3L1 Adipocytes WM1341D CAA A375 CAA WM9 CAA Hs294T CAA

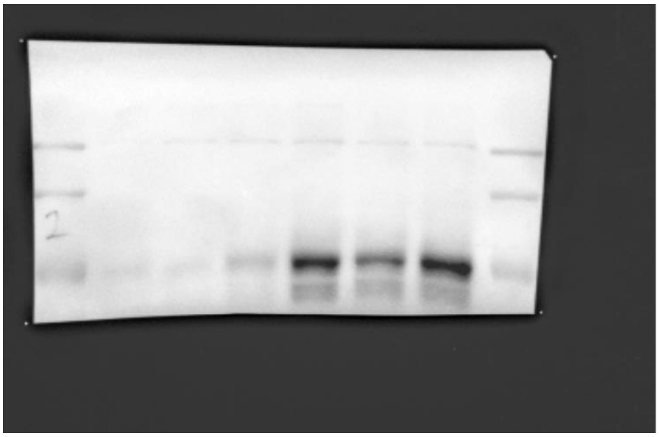

3T3L1 Adipocytes WM1341D CAA A375 CAA WM9 CAA Hs294T CAA

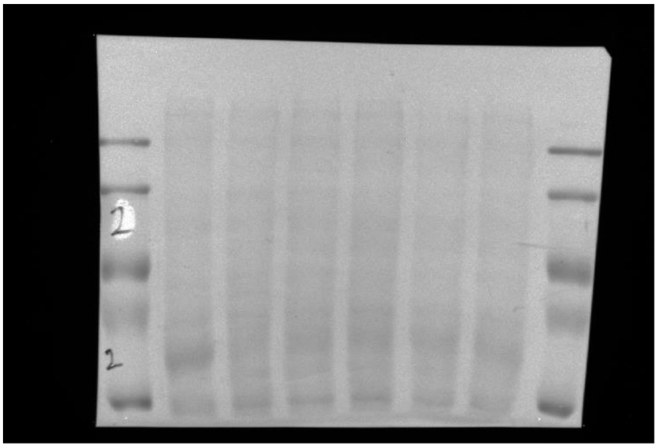

TGFβRIII

TGFβRIII Repetition 1 Ponceau staining

3T3L1 Adipocytes WM1341D CAA A375 CAA WM9 CAA Hs294T CAA

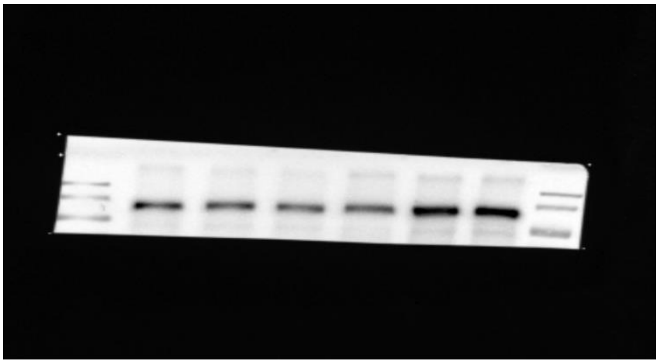

3T3L1 Adipocytes WM1341D CAA A375 CAA WM9 CAA Hs294T CAA

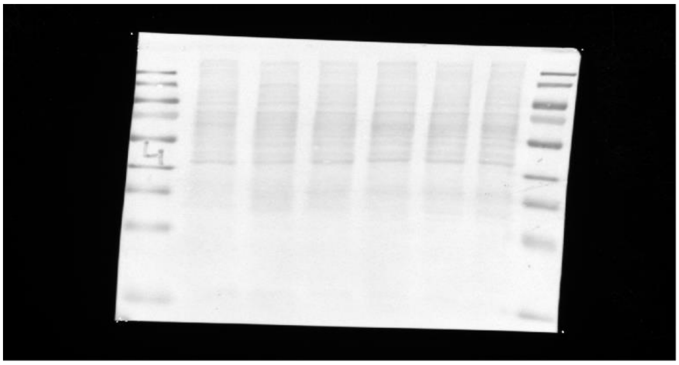

TGFβRIII Repetition 2 Ponceau staining

3T3L1 Adipocytes WM1341D CAA A375 CAA WM9 CAA Hs294T CAA

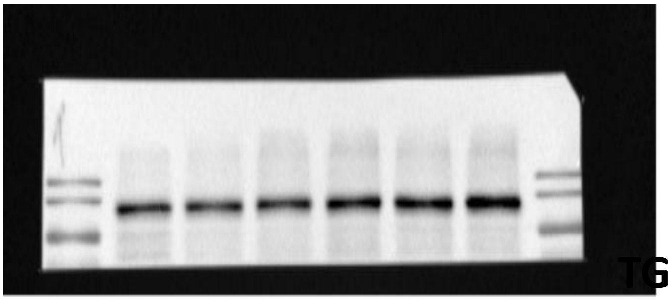

3T3L1 Adipocytes WM1341D CAA A375 CAA WM9 CAA Hs294T CAA

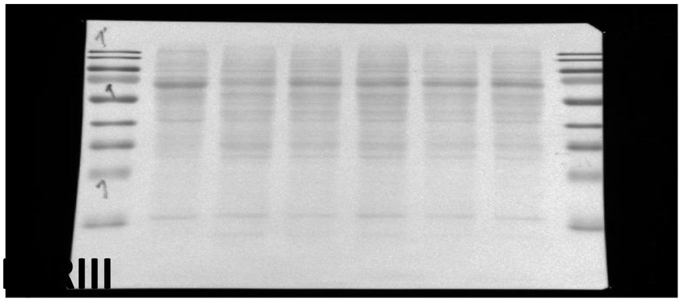

TG[illegible]RIII

TGFβRIII Repetition 3 Ponceau staining

3T3L1 Adipocytes WM1341D CAA A375 CAA WM9 CAA Hs294T CAA

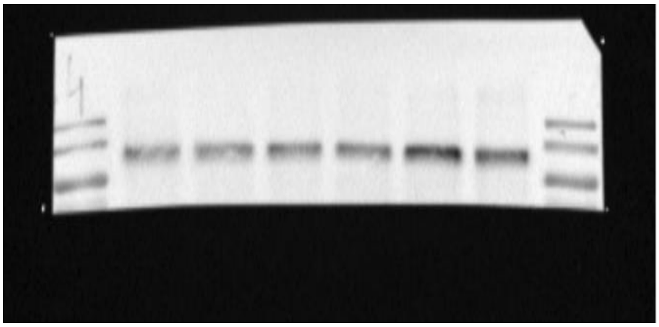

3T3L1 Adipocytes WM1341D CAA A375 CAA WM9 CAA Hs294T CAA

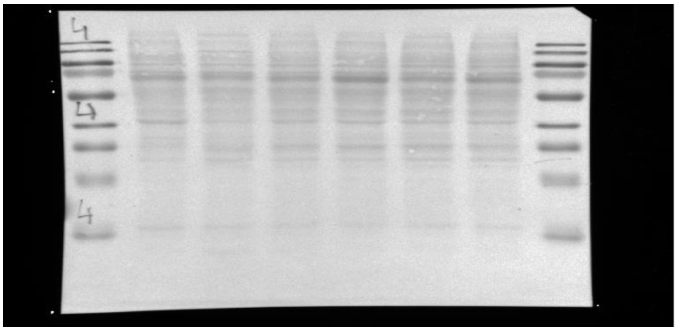

WIMENTIN

WIMENTIN Repetition 1 Ponceau staining

3T3L1 Adipocytes WM1341D CAA A375 CAA WM9 CAA Hs294T CAA

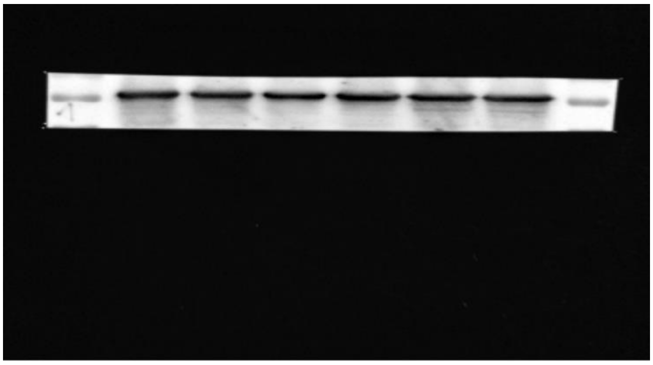

3T3L1 Adipocytes WM1341D CAA A375 CAA WM9 CAA Hs294T CAA

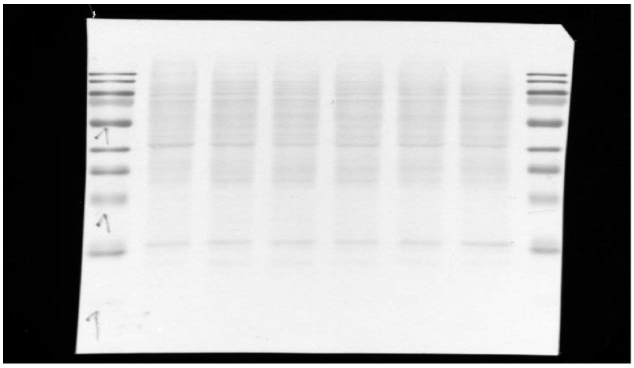

WIMENTIN Repetition 2 Ponceau staining

3T3L1 Adipocytes WM1341D CAA A375 CAA WM9 CAA Hs294T CAA

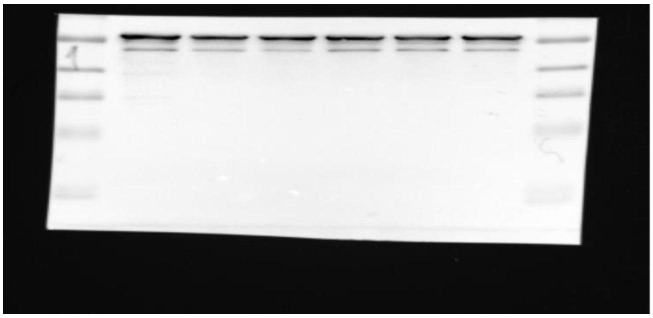

3T3L1 Adipocytes WM1341D CAA A375 CAA WM9 CAA Hs294T CAA

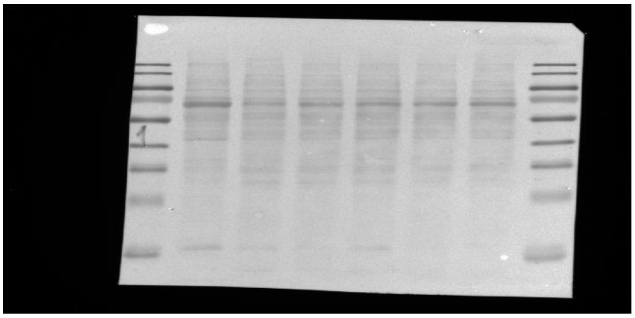

WIMENTIN Repetition 3 Ponceau staining

3T3L1 Adipocytes WM1341D CAA A375 CAA WM9 CAA Hs294T CAA

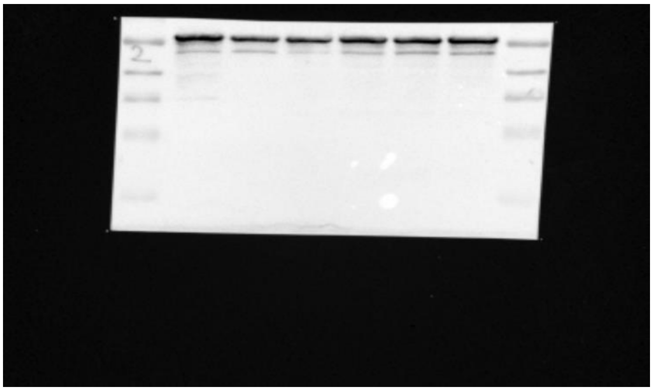

3T3L1 Adipocytes WM1341D CAA A375 CAA WM9 CAA Hs294T CAA

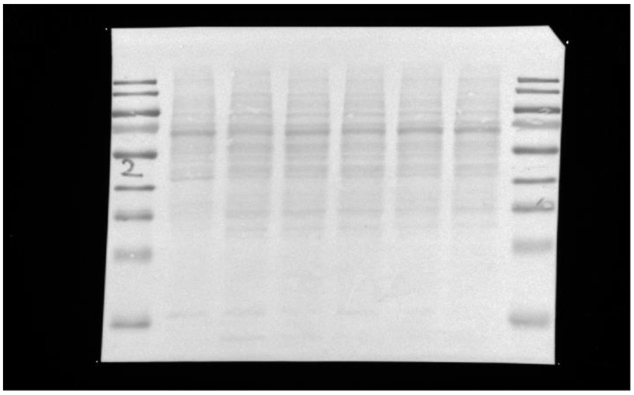

WIMENTIN Repetition 4 Ponceau staining

3T3L1 Adipocytes WM1341D CAA A375 CAA WM9 CAA Hs294T CAA

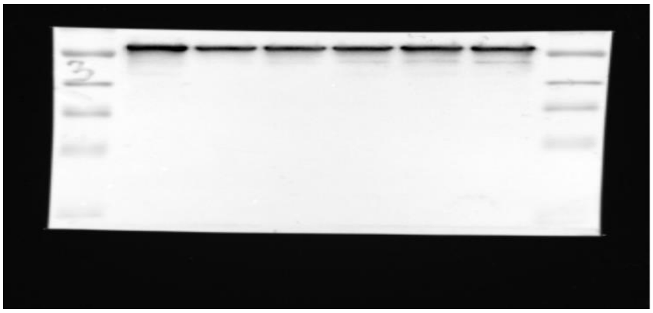

3T3L1 Adipocytes WM1341D CAA A375 CAA WM9 CAA Hs294T CAA

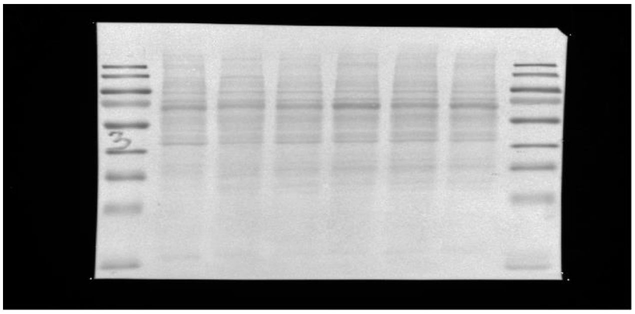

Supplement: Supplementary file 1 — Additional file 1: The nitroceluluse membranes on which are visualized bands corresponding to selected proteins (ERK1/2, pERK1/2, FASN, STAT3, pSTAT3, TGFßRIII, vimentin) or Ponceau S staining. [file 11658_2023_476_MOESM1_ESM.pdf]
